# Rapid Synthesis of MIV-Based Multimetal–Organic Frameworks for Photopolymerization of Methyl Methacrylate under Visible Light

**DOI:** 10.1021/acsami.6c00818

**Published:** 2026-03-16

**Authors:** Mateusz Adam Baluk, Magdalena Miodyńska-Melzer, Joanna Drzeżdżon, Kostiantyn Nikiforow, Tomasz Klimczuk, Krzysztof Matus, Stefania Zappia, Adriana Zaleska-Medynska

**Affiliations:** † Department of Environmental Technology, Faculty of Chemistry, 427055 University of Gdańsk, Wita Stwosza 63, Gdansk 80-308, Poland; ‡ 119463 Institute of Physical Chemistry, Polish Academy of Science, Kasprzaka 44/52, Warsaw 01-224, Poland; § Faculty of Applied Physics and Mathematics, Gdansk University of Technology, Narutowicza 11/12, Gdansk 80-233, Poland; ∥ Advanced Materials Center, Gdansk University of Technology, Gdansk 80-233, Poland; ⊥ 49569 Silesian University of Technology, Gliwice 44-100, Poland; # 9327 Istituto di Scienze e Tecnologie Chimiche “Giulio Natta” (SCITEC) of the Consiglio Nazionale delle Ricerche (National Research Council - CNR), via Alfonso Corti 12, Milano 20133, Italy

**Keywords:** multimetal MOF, visible light, photopolymerization, MMA, polymers

## Abstract

Metal–organic frameworks (MOFs) are porous materials that have attracted increasing interest for photocatalytic applications. Conventional solvothermal synthesis of photoactive MOFs typically requires high-pressure reactors and time-consuming preparative protocols. This study reports the preparation of Ti-, Zr-, and/or Hf-based MOFs using a hot injection approach. Among the prepared materials, NH_2_-UiO-66 (Zr/Hf) exhibited high photocatalytic activity for the polymerization of methyl methacrylate under visible light irradiation and under monochromatic visible light at a wavelength of 430 nm (xenon lamp or light-emitting diode). The ability to produce these materials using simple, low-pressure equipment makes this method a promising route for process scale-up and cost reduction. The obtained MOFs demonstrated high photostability, maintaining nearly unchanged activity over three photocatalytic cycles and during long-term operation (44 h). Moreover, the polymer products were analyzed via size exclusion chromatography and complementary analytical techniques, thereby elucidating the photopolymerization process under different irradiation conditions and reaction times.

## Graphical abstract

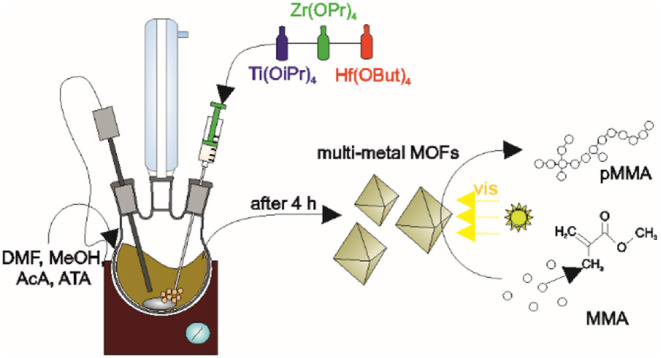

## 1 Introduction

Global demand for metal, ceramic, wood, glass, and paper products has led to the search for alternative materials, resulting in considerable research interest in polymeric particles.^1,2^ These materials are composed of long, straight, or branched organic structures with controllable properties, such as shape, degree of branching, size,^3^ and surface functionalization.^4^ One commonly used polymer is poly­(1-(methoxycarbonyl)-1-methylethyl) (PMMA), which is formed through the chain-growth polymerization of its monomer, methyl methacrylate (MMA).^5^ PMMA is used in fields such as biomedicine,^6^ transportation,^7^ and textiles^8^ owing to its unique transparent, thermoplastic, and amorphous properties, biocompatibility, and ability to dissolve in organic solvents. Although global production of this polymer exceeds 4 million tons,^9^ conventional synthesis methods struggle with problems such as heat dissipation, gelation, and side reactions. Photopolymerization is an efficient monomer-bonding reaction initiated by ultraviolet (UV) or visible (Vis) light^10^ that can proceed via free radical photopolymerization, reversible-deactivation radical polymerization, or surface activation.^10^ Among them, free radical photopolymerization is the most common mechanism in semiconductor-based systems, which occurs via direct activation of the monomer,^11^ indirect activation with a co-initiator,^12^ or surface polymerization.^13^

Considerable research is currently focused on the use of well-known semiconductors as photocatalysts for MMA polymerization. These materials are mainly based on ZnO,^14^ TiO_2_,^15^ Fe_2_O_3_,^16^ CdS,^17^ or g-C_3_N_4_ ^18^ and typically require high-energy, costly UV irradiation or special amine co-initiators to catalyze the reaction, as shown in Table S1. An excellent alternative may be a new group of materials that are responsive to light, particularly visible light: metal–organic frameworks (MOFs).

Although several types of MOFs can reportedly polymerize MMA, only a few have demonstrated activity via photoinitiation. Using a different approach, MOF-907 (Fe) was shown to catalyze MMA polymerization in the presence of ethyl-α-bromophenylacetate or ethyl-α-bromoisobutyrate (as co-initiators) under microwave-assisted conditions.^19^ Further, IRMOF-3@MOF-5 ^20^ and IrBPY-MOL^21^ have been utilized to catalyze atom transfer radical polymerization.

To date, only a few zinc- and zirconium-based MOFs, such as NNU-35,^22^ ZIF-8,^23^ and NNU-28,^24^ have been utilized in typical photopolymerization reactions driven by UV or visible light. One representative group of photoactive MOFs is UiO-66–based materials, which exhibit activity in photocatalytic reactions such as CO_2_ photoconversion,^25^ hydrogen photogeneration,^26^ and decomposition of pollutants,^27^ as well as in catalytic reactions including esterification,^28^ Suzuki–Miyaura coupling, nitroarene reduction,^29^ and selective hydrogenation.^30^ UiO-66–based MOFs are considered promising photocatalysts for MMA photopolymerization owing to their favorable electronic structure and relatively small optical band gap, enabling excitation by low-energy, cost-effective visible light.

This study proposes a rapid method for synthesizing photoactive MOFs via hot injection molding of NH_2_-UiO-66-structured materials with mono- or bimetallic secondary building units based on zirconium (Zr) and/or hafnium (Hf). The structural, surface, and optical properties of the obtained MOFs were characterized. Attempts to obtain crystalline materials based on titanium (Ti), Zr, and/or Hf yielded only amorphous materials. The metals chosen for modification were selected according to the photoactivity of UiO-66–based materials containing these ions but synthesized using conventional and well-established methods.^31,32^ The approach offers scalability and easy MOF modifiability compared to commonly used methods. Furthermore, bimetallic NH_2_-UiO-66–derived MOFs are reported as photocatalysts for light-induced MMA photopolymerization. This study advances the current understanding of this process by providing systematic, experimentally supported insights on the effect of irradiation type (e.g., visible, solar, monochromatic, or light-emitting diode [LED]) on PMMA production (e.g., yield, polydispersity index [PDI], and molecular weight [*M*_w_]).

## 2 Experimental Section

### 2.1 Materials

*N,N*-Dimethylformamide (DMF, p.a.), chloroform (p.a.), and sodium sulfate (Na_2_SO_4_, p.a.) were purchased from POCh (Poland). Methanol (MeOH, p.a.) and acetic acid (AcA, 99.9%) were purchased from STANLAB (Poland). 2-Aminoterephthalic acid (ATA, 98%), tetrahydrofuran (THF, > 99%), titanium­(IV) isopropoxide (Ti­(OiPr)_4_, 98%), zirconium­(IV) propoxide (Zr­(OPr)_4_, 70% in 1-propanol), and hafnium­(IV) n-butoxide (Hf­(OBut)_4_, 99%), 2,5-dihydroxybenzoic acid (2,5-DHB, 99.5%), butylated hydroxytoluene (BHT, 99%), ethyl-α-bromophenylacetate (EBPA, 97%) were purchased from Merck (Germany). Nafion solution D520 was purchased from Ion Power (Germany). Ethylenediaminetetraacetic (99.5%, EDTA) was purchased from Acros Organic (Belgium). Dimethyl sulfoxide (DMSO) was purchased from Eurochem BGD (Poland). DMF and DMSO were stored in bottles containing silica gel to adsorb any water and other contaminants.

### 2.2 Sample Preparation

The MOF materials were obtained using the modified hot injection method previously described by Baluk et al.^33^ The MOF production scheme is illustrated in Figure 1. Briefly, 1.086 g of ATA was dissolved in a mixture of 36 mL of DMF, 4 mL of MeOH, and 4 mL of AcA and heated to 125 °C in a three-neck round-bottomed flask equipped with a reflux condenser and a heating jacket. After boiling for 10 min, the appropriate amounts of Ti^4+^, Zr^4+^, and/or Hf^4+^ precursor (at room temperature in 25 °C) were added to the mixture, resulting in the formation of the corresponding monometallic, bimetallic, and trimetallic materials, as shown in Table S2. The mixture was vigorously stirred and continuously heated to the boiling point for 4 h. Subsequently, the material was centrifuged (12,000 rpm), washed with DMF (3×) and MeOH (3×), and dried under dynamic vacuum at 200 °C. The Zr@Hf/BDC-NH_2_ sample was obtained by postsynthetic modification of Hf/BDC-NH_2_. For this purpose, 1 g of Hf/BDC-NH_2_ was resuspended in 25 mL of DMF containing 4.3 mmol of ZrCl_4_ and heated at 120–125 °C (in boiling points) for 24 h, after which the material was washed and dried as described above.

**Figure 1:**
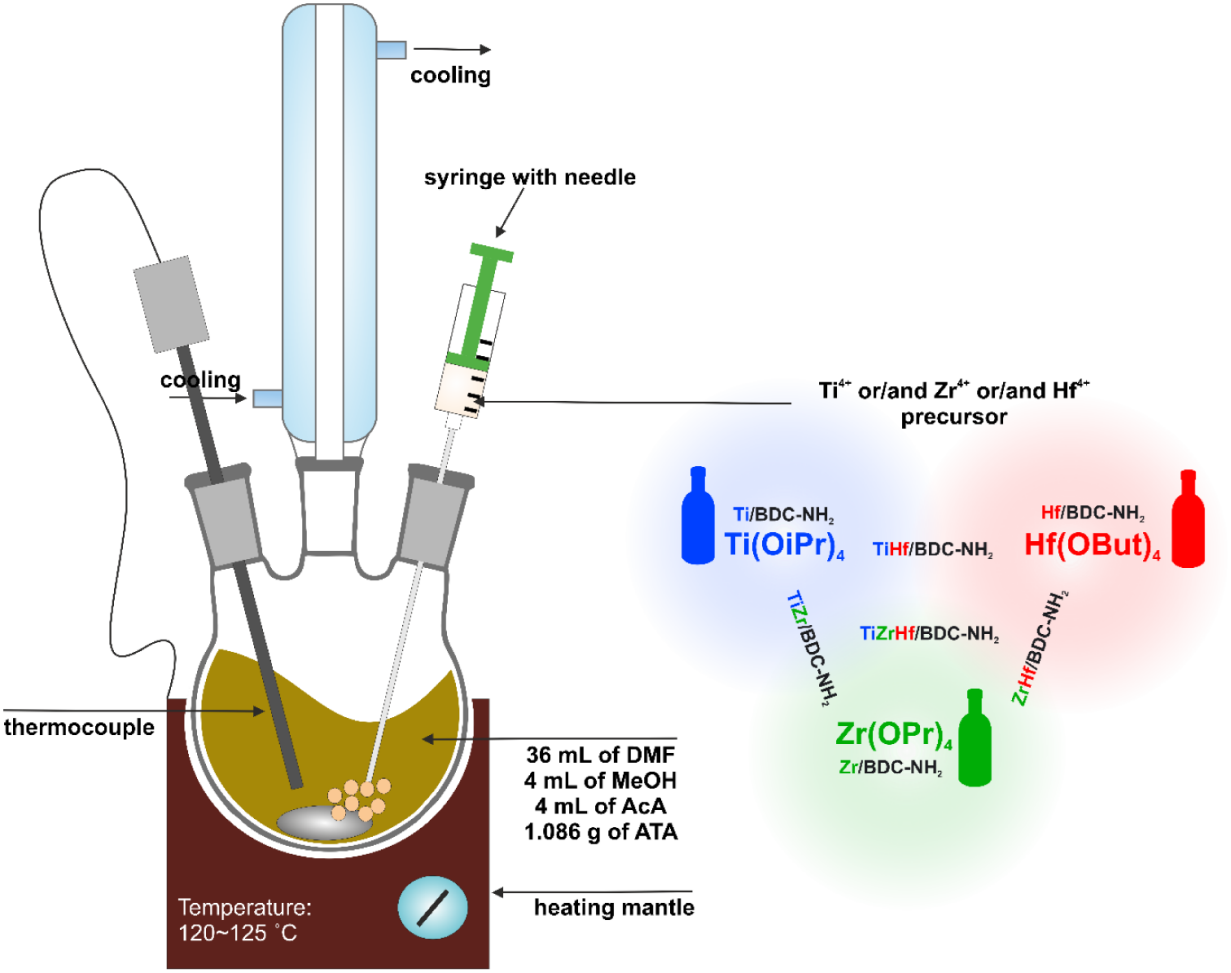
Synthesis scheme for multimetal/BDC-NH_2_ materials.

### 2.3 Sample Characterization

Morphological analyses were conducted via scanning electron microscopy (SEM) using a JEOL JSM-7610F scanning electron microscope and transmission electron microscopy (TEM) coupled with energy-dispersive X-ray spectroscopy (EDS) using an FEI S/TEM TITAN 80–300 high-resolution transmission electron microscope.

Powder X-ray diffraction (pXRD) was conducted at room temperature on powdered samples using a Bruker D8 Focus diffractometer equipped with Cu Kα (λ = 1.54 Å) radiation and a LynxEye XE-T detector in the 2θ range of 3°–70°.

Photocurrent (PC) experiments were performed using a Metrohm Autolab PGSTAT204 potentiostat with Pt wire as the counter electrode, an Ag/AgCl electrode as the reference electrode, and photocatalyst powder deposited on fluoride tin oxide (FTO) glass as the working electrode. The light source was a 150 W xenon lamp from Hamamatsu Photonics (Japan), and the electrolyte was an aqueous solution of sodium sulfate (0.1 M, 15 mL). The working electrode was prepared by depositing 50 μL of photocatalyst suspension on a 20 × 5 mm^2^ FTO glass plate. The photocatalyst suspension contained 5 mg of MOF material ultrasonically dispersed in 1 mL of water, 0.375 mL of isopropyl alcohol, and 30 μL of Nafion solution.

Diffuse reflectance UV–Vis spectra were collected in the 250–700 nm range using a Shimadzu UV-2600 spectrophotometer equipped with an integrating sphere, employing BaSO_4_ as a nonabsorbing reference. The optical band gap energies of the MOF materials were determined using the Tauc plot method.

Thermogravimetric analysis (TGA) was performed within the temperature range of 28 °C–1000 °C using a PerkinElmer TGA 8000 instrument at a heating rate of 10 °C·min^–1^ under N_2_ flow (10 mL·min^–1^).

FT-IR spectra of MOFs were collected in the spectral range of 480–4500 cm^–1^ (diffuse reflectance mode) using a Thermo Scientific Nicolet iS10 spectrometer (8 cm^–1^ resolution) at room temperature. The samples consisted of 10 wt % photocatalyst dispersed in KBr. FT-IR spectra of polymers were obtained in the spectral range of 4500–500 cm^–1^ using a Thermo Scientific Nicolet iS5 Transmission iD1 (0.12 cm^–1^ resolution) and the KBr pellet method.

X-ray photoelectron spectroscopy (XPS) was performed using an ULVAC-PHI 5000 VersaProbe II hybrid spectrometer equipped with monochromatic Al Kα radiation (hν = 1486.6 eV). The scanning X-ray source was operated at a power of 25 W, an acceleration voltage of 15 kV, with a spot size of 100 μm, a pass energy of 23.5 eV, and an energy step of 0.1 eV. The obtained XPS spectra were analyzed using CasaXPS software, and the Shirley background and Gaussian–Lorentzian peak shapes were employed for the deconvolution of all spectra.

Matrix-assisted laser desorption/ionization time-of-flight mass spectrometry (MALDI-TOF-MS) was performed using a BRUKER Autoflex maX spectrometer featuring a SmartBeam-II solid-state laser. Samples were prepared using the dried-droplet method, whereby a 1:1 volumetric mixture of the analyte and matrix solution was deposited onto the target plate. The matrix solution consisted of 20 mg/mL 2,5-DHB dissolved in TA30 (a solution of 30% acetonitrile and 70% aqueous 0.1% TFA). External calibration was conducted using Bruker’s standard calibration mixture, spanning the mass range of 0–2000 Da.

Size exclusion chromatography (SEC) was performed using two PL polypore columns using THF containing 0.05% BHT as the eluent and a flow rate of 0.8 mL·min^–1^. The column temperature was set at 35 °C, with an injection volume of 150 μL. The average sample concentration was approximately 2 mg·mL^–1^. Samples were completely dissolved in THF with 0.05% BHT, with no visible insoluble fraction, aggregates, or precipitates, and filtered using 0.2 μm PTFE membranes. For calibration, a relative curve was determined based on the log­(M) = *f*(V) relationship, where M denotes the molecular weight and V represents the elution volume. A third-order polynomial calibration curve was prepared with 20 narrow molecular weight distribution (MWD) polystyrene standards in the peak molecular weight (M_p_) range of 1,670,000–162 g·mol^–1^ (styrene monomer). This calibration curve was employed for the molecular mass determination of samples. Data acquisition and processing were performed using Waters HPLC/SEC Empower Pro 1.0 chromatographic software.

^1^H and ^13^C NMR spectra were recorded using a Bruker Avance III 500 (500/125 MHz) spectrometer at 298 K, with CDCl_3_ used as the solvent.

### 2.4 Photocatalytic Properties

The photocatalytic properties of the MOF materials were investigated using a model MMA photopolymerization reaction in a sealed quartz tube (volume = 30 mL, wall thickness = 1 mm) equipped with stirrer. Briefly, 12 mg of MOF material was dispersed in 4.2 mL of DMF, followed by the addition of 1.2 mL of MMA and 10 μL of ethyl-α-bromophenylacetate as a co-initiator. The tube was sealed with a rubber septum. To remove excess oxygen, the reactor was purged with an N_2_ stream (12 dm^3^·h^–1^) for 1 min. MMA photopolymerization was initiated using a 1000 W xenon lamp (Oriel, USA) equipped with optical filters. The effects of different irradiation types (λ > 420 nm, λ > 400 nm, and sunlight) on the efficiency of the process were investigated using different optical filters (GG420, GG400, and AM 1.5 G, respectively) over 2–44 h at room temperature. The efficiency of PMMA synthesis using ZrHf/BDC-NH_2_ under monochromatic radiation was investigated using a xenon lamp at λ = 400, 420, 430, 440, 450, and 460 nm for 24 h. For comparison, the process was also performed under LED radiation at λ = 430 nm for 4 h. To determine the roles of electrons and holes, a photopolymerization experiment was performed using ZrHf/BDC-NH_2_ for 4 h using visible light (λ > 420 nm) under the conditions described above but with DMSO and EDTA (5 mM each) added as electron and hole scavengers, respectively.

After each photocatalytic reaction, the DMF solution with PMMA was separated from the photocatalyst via centrifugation. PMMA was then precipitated using a mixture containing 18 mL of MeOH and 2 mL of deionized water (Figure 2), separated via filtration, and dried at room temperature for 24 h.

**Figure 2:**
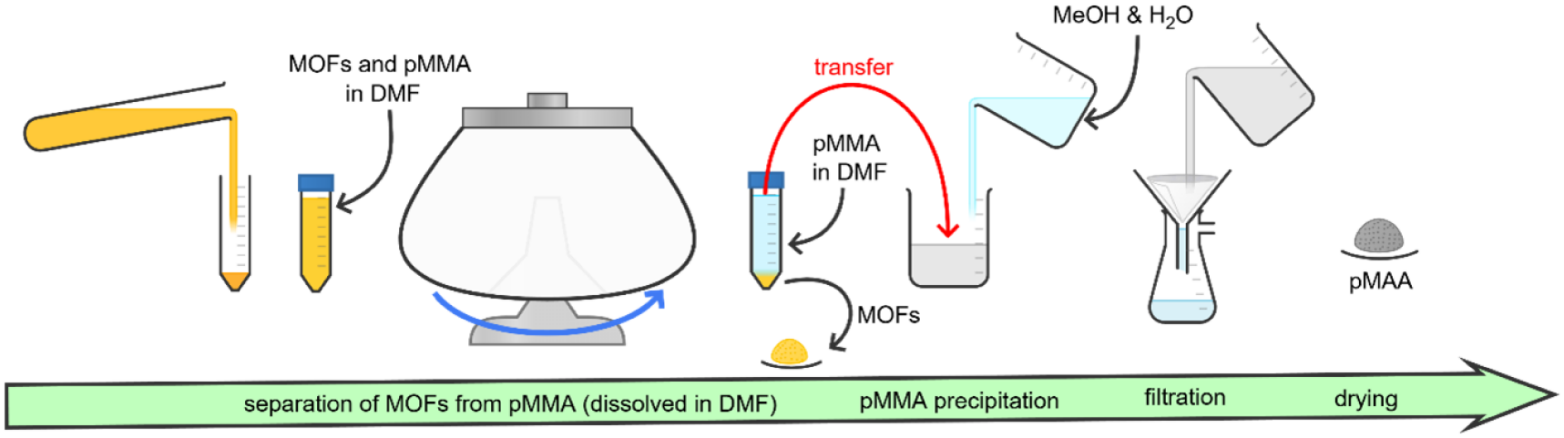
Schematic diagram of MOF recovery process and precipitation of pMAA.

To examine photocatalyst stability, the photopolymerization process was conducted three times using the same parameters. After each cycle, the material was centrifuged, washed with fresh DMF (3×) and MeOH (3×) to remove PMMA and MMA residues, and dried under dynamic vacuum at 200 °C for 12 h. The purified powder was used in the next cycle.

## 3 Results

MOF materials were obtained by injecting organic precursors of Ti, Zr, and/or Hf into a boiling solution of ATA in DMF, MeOH, and AcA. The proposed method is compared to solvothermal and microwave methods for synthesizing UiO-66–based materials in Table 1. The hot injection method shortens the reaction time to approximately 4 h and provides good process control, including the ability to precisely control the reaction time, add modifiers during synthesis, and monitor reaction progress. Additional advantages include lower energy consumption and simpler instrumentation, as the reaction can be performed in a round-bottomed flask under reflux, eliminating the need for high-pressure reactors. The proposed method also has greater scale-up potential than solvothermal and microwave methods, making it a practical and economical solution for synthesizing UiO-66–based materials and other MOFs.

**Table 1:** Comparison of Methods for Synthesizing UiO-66–Based Materials

Criteria	Solvothermal method	Microwave method	Hot injection method
Rate of reaction	Long (24 h)	Short (15 min)	Short (4 h)
Synthesis temperature	120 °C	120 °C	∼120 °C
Process control	Difficult to precisely control temperature and pressure	Enables precise control of heating time and power	Enables precise control of heating time and possibility of adding multiple modifiers during synthesis
Check reaction progress	Impossible	Possible in special reactors with cameras	Possible
Energy efficiency	Large energy losses associated with heating the furnace and long process times	High energy needed to produce microwaves, but low energy loss associated with short process time	Low energy required to heat the reaction and low energy losses associated with short process time
Equipment limitations	High-pressure reactor	High-pressure reactor and consumables (flasks and caps)	Laboratory round-bottomed flask and need for continuous cooling during reflux using water or refrigerant
Scale	Need to adapt synthesis to small-volume reactors	Challenging to scale up due to high device cost and user safety	Potential for scale-up
Ref	Li et al.^34^	Taddei et al.^35^	This work

### 3.1 Physicochemical Properties of Samples

SEM and TEM images of the obtained MOF materials are presented in Figures 3 and S1–S7, and their size distributions are shown in Figure S8. Selected properties, such as particle size and shape, BET surface area, and CO_2_ uptake, are presented in Table S2. Pristine Zr/BDC-NH_2_ exhibited an octahedral-type shape with a particle size of 51 nm (Figures S1 and S4) and a similar surface area (473 m^2^·g^–1^) and CO_2_ sorption (1.30 mmol·g^–1^) as previously obtained Ti/BDC-NH_2_ (NH_2_-MIL-125 (Ti)^33^). Its counterpart, Hf/BDC-NH_2_, exhibited a significantly smaller particle size (∼19 nm) and irregular shape, although some particles were similar to typical octahedral UiO-66 particles (Figures S1 and S5). The specific surface area and CO_2_ sorption of Zr/BDC-NH_2_ dropped to 278 m^2^·g^–1^ and 0.6 mmol·/g^–1^, respectively. The MOF materials based on Zr^4+^, Ti^4+^, and/or Hf^4+^ (TiZr/BDC-NH_2_ and ZrHf/BDC-NH_2_, respectively) exhibited octahedral-type shapes with particle sizes of 29 and 43 nm, respectively (Figures 3 and S6). Notably, the remaining multimetal MOF materials based on Ti^4+^ and another metal (TiHf/BDC-NH_2_ and TiZrHf/BDC-NH_2_) typically exhibited irregular shapes with particle sizes of 19 and 40 nm, respectively (Figures 3 and S2). Their specific surface areas ranged from 151 to 392 m^2^·g^–1^ and CO_2_ sorption values ranged from 0.39 to 0.91 mmol·g^–1^, with ZrHf/BDC-NH_2_ exhibiting the highest values for both parameters. In addition, the MOF material obtained via ion metathesis of Hf^4+^ to Zr^4+^ (Zr@Hf/BDC-NH_2_) exhibited a significantly different specific surface area (76 m^2^·g^–1^) and CO_2_ sorption (0.28 mmol·g^–1^), and a slightly larger particle size, than the material from which it was obtained (Hf/BDC-NH_2_) (Figures S3 and S7). TEM-EDS analysis of ZrHf/BDC-NH_2_ (Figure S6) and Zr@Hf/BDC-NH_2_ (Figure S7) revealed that Zr (green) and Hf (purple) were evenly distributed throughout the particles.

**Figure 3:**
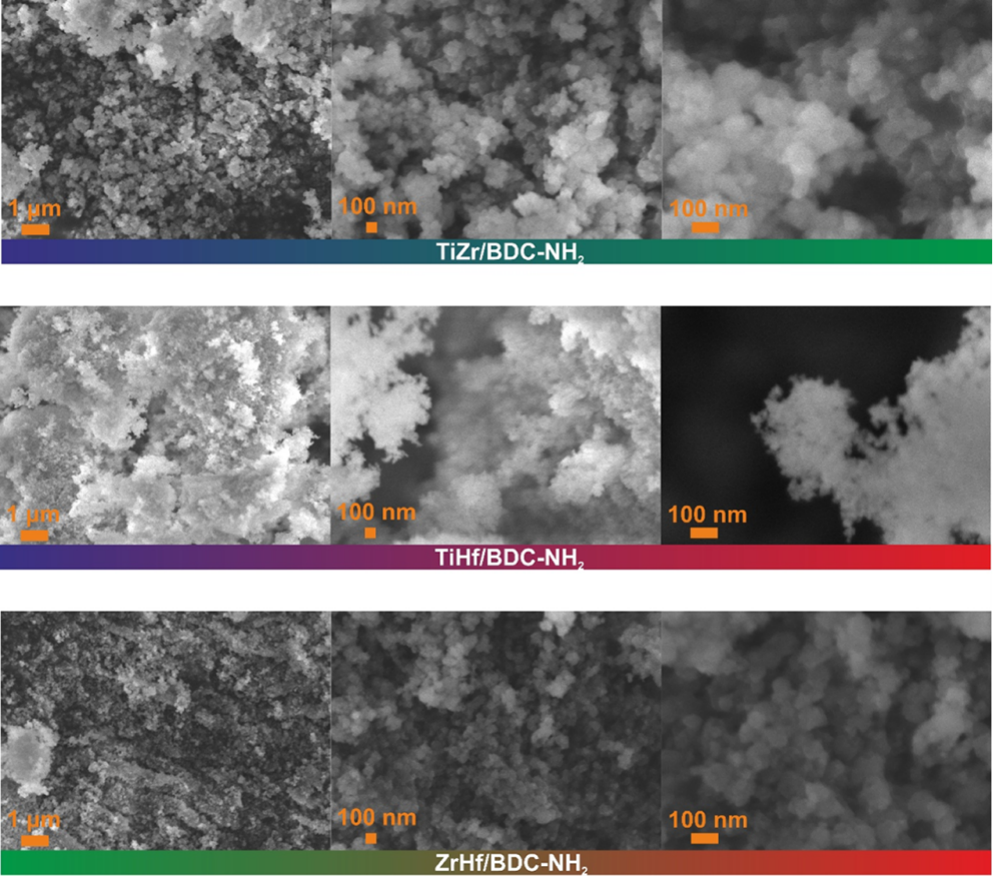
SEM images of TiZr/BDC-NH_2_, TiHf/BDC-NH_2_, and ZrHf/BDC-NH_2_.

As previously reported, the material obtained via hot injection of Ti^4+^ and ATA resulted in well-crystallized NH_2_-MIL-125 (Ti) (Figure 4a). The same reaction using Zr^4+^ or Hf^4+^ resulted in materials with reflections consistent with NH_2_-UiO-66 (Zr)^36^ or NH_2_-UiO-66 (Hf),^37^ although a substantial deterioration in crystallinity was observed for the Hf-containing material (Figure 4a), which was attributed to its very small particle size, as previously mentioned (Figure S5). Among bimetallic materials (molar ratio of 1:1), only ZrHf/BDC-NH_2_ exhibited typical reflections for NH_2_-UiO-66 (Zr/Hf) particles, while attempts to combine other metals (i.e., TiHf/BDC-NH_2_ and TiZr/BDC-NH_2_) resulted in the formation of amorphous materials (Figure 4b). Notably, although the attempt to obtain trimetallic MOFs based on ATA resulted in the formation of considerably amorphous products, slight reflections at 7.5°, 8.6°, 12.3°, 17.3°, and 26° could only be attributed to the UiO-66 structure (Figure S9). Modification of Hf/BDC-NH_2_ with Zr^4+^ did not substantially change the crystal structure of the material, which remained slightly amorphous (Figure S10).

**Figure 4:**
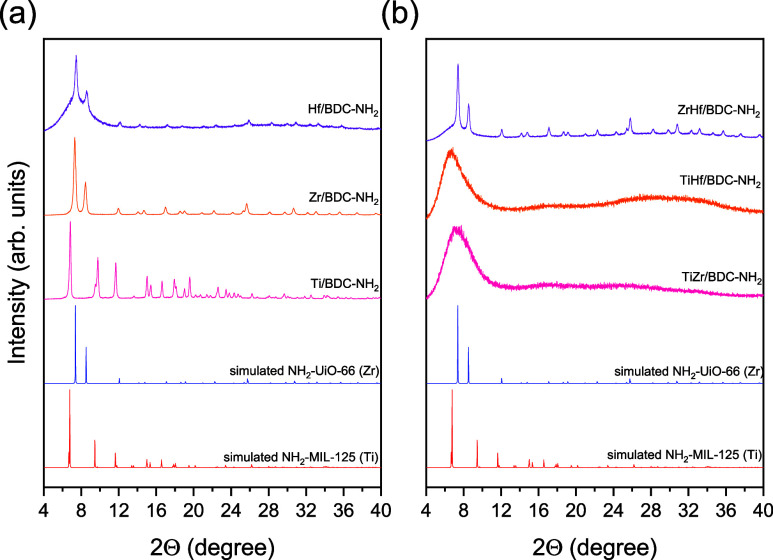
XRD analysis of (a) pristine MOFs: Ti/Zr/Hf/BDC-NH_2_ and (b) TiZr/TiHf/ZrHf/BDC-NH_2_ with simulated plots from VESTA Software^38^ (Card Nos. 7211159 for NH_2_-MIL-125 (Ti) and 4512072 for NH_2_-UiO-66 (Zr) from Crystallography Open Database^39^).

The FTIR spectra of the MOF materials are presented in Figure S11. The characteristic absorption bands of ATA were observed in all spectra owing to its use as a ligand in MOF synthesis, including bands at 3358 and 3480 cm^–1^, which were ascribed to symmetric and asymmetric N–H stretching vibrations, respectively.^40^ Bands at 1571 and 1658 cm^–1^ were attributed to the synergistic vibrations of carboxyl groups and metal cations (Zr^4+^, Hf^4+^, and Ti^4+^), respectively.^41^ These signals were also present in the FTIR spectra of mixed-cation MOFs, although with slightly reduced intensity. A bending vibration associated with the N–H group was detected at 1496 cm^–1^.^41^ The bands at approximately 1445, and 1382 cm^–1^ were ascribed to the stretching vibrations of C–N and C–C bonds, respectively.^42^ The presence of amino groups in the MOF materials was further confirmed by the bands at 769 cm^–1^ (N–H) and 1257 cm^–1^ (C–N)^43^ Additionally, the bands at 580 and 660 cm^–1^ were attributed to C–H and O–H vibrations, respectively.^42^ Bending vibrations ascribed to metal–oxygen (M–O) bonds were evident below 500 cm^–1^.^44^ Taken together, these spectral features confirmed the organometallic framework structure of the synthesized MOFs.

TGA (Figure S12) revealed that Ti/BDC-NH_2_ and Zr/BDC-NH_2_ exhibited comparable thermal stability up to 200 °C, while Hf/BDC-NH_2_ remained stable up to approximately 300 °C. All multimetal MOF materials displayed a weight loss between 280 and 300 °C, followed by a sharp decrease in weight between 400 and 600 °C, characteristic of MOF decomposition into metal oxides.^44,45^

XPS analysis confirmed the presence of carbon, nitrogen, oxygen, and corresponding metals in the MOF materials. The high-resolution XPS spectra of these elements for ZrHf/BDC-NH_2_ and Zr@Hf/BDC-NH_2_ are presented in Figure 5. The C 1s peak was successfully fitted with three synthetic components, corresponding to C–C (284.8 eV), C–N (286.5 eV), and COOH (288.9 eV) bonds. A single chemical state was observed for nitrogen (N 1s 399.8 eV), as expected for the chemical structure of NH_2_-UiO-66. The O 1s region was fitted with three peaks, which were assigned to O–Zr–O (530 eV), O–Zr–C (531.1 eV), and C–O (532.4 eV) bonds, respectively.^46,47^ The Ti 2p spin orbit doublet was observed at 459 eV (Ti 2p 3/2) and 464.7 eV (Ti 2p 1/2) (Figure S13). The positions and peak separation suggested that Ti was present in the 4+ oxidation state.^48^ The peaks at approximately 17.5 and 19.1 eV corresponded to a doublet peak of Hf 4f_7/2_ and Hf 4f_5/2_, which could be assigned to Hf in the 4+ oxidation state considering the binding energies.^49,50^ The Zr 3d spectra exhibited peaks at 182.8 and 185.2 eV, which were attributed to Zr 3d 5/2 and Zr 3d 3/2, respectively, suggesting that Zr could also be assigned to the 4+ oxidation state.^51,52^ Unlike Ti and Hf, the deconvoluted Zr 3d spectra consisted of two doublet peaks—the first set at 182.2 eV (Zr 3d 5/2) and 184.6 eV (Zr 3d 3/2), and the second set at 183.2 and 185.6 eV. This spectrum fitting aligned with that of similar materials.^53^ The lower energy doublet could be assigned to Zr in the cluster node (Zr–O and Zr–OH), while the higher energy doublet was assigned to Zr bound to carboxyl groups.^51,52^

**Figure 5:**
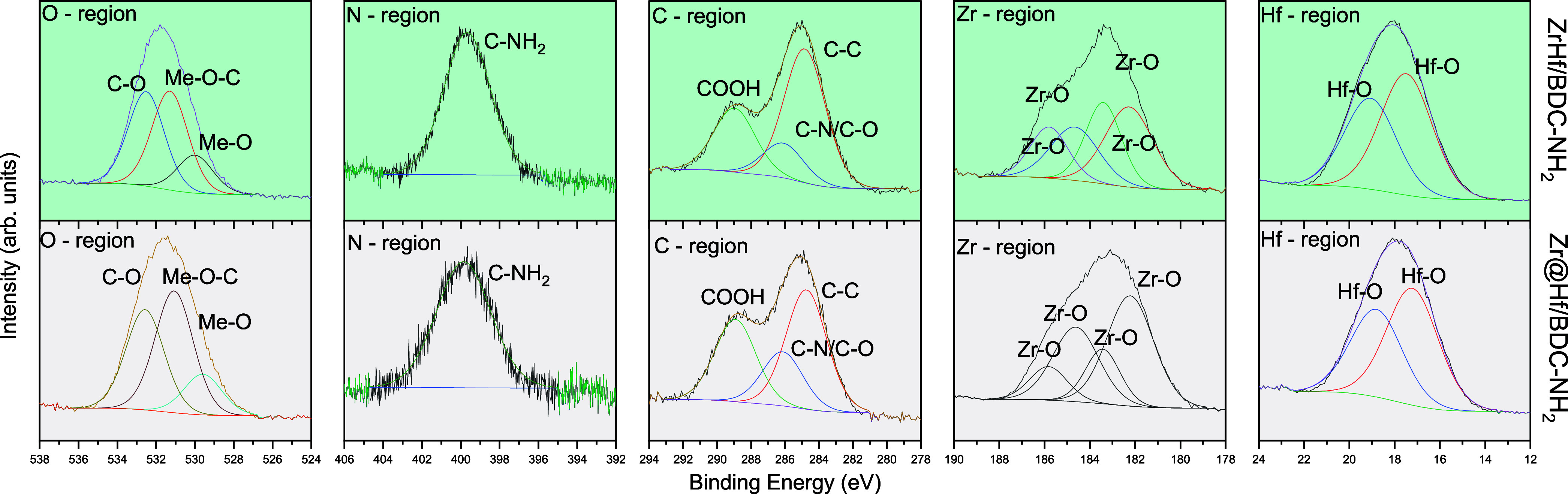
Detailed analysis of elemental O, N, C, Zr, and Hf for ZrHf/BDC-NH_2_ and Zr@Hf/BDC-NH_2_ (Me = Zr or Hf).

Figure S14 presents the photocurrent response of the MOF materials recorded under intermittent UV–Vis illumination (every 50 s). An immediate increase in current density was observed after the light was turned on, indicating effective generation and separation of electron–hole pairs. The signal returned to its initial value after the light was turned off, confirming the reversible nature of the process. The largest current response (approximately 0.54 μA) was observed for ZrHf/BDC-NH_2_.

The UV–Vis/DRS spectra of the MOF materials are presented in Figure S15a, demonstrating comparable spectral profiles, with absorption in the UV and visible regions reaching up to approximately 550 nm. Notably, TiZr/BDC-NH_2_ and TiHf/BDC-NH_2_ exhibited the most pronounced red shifts in the absorption edge toward longer wavelengths among the obtained MOF materials.

The highest occupied crystalline orbital (HOCO) position of the MOF materials was determined based on the low-binding-energy region of the XPS spectra (Figure S16), and the optical gap was calculated based on the DRS spectra and Tauc plots (Figure S15b). Based on these determinations and calculations, a band diagram of the obtained MOF materials is presented in Figure S17, illustrating the HOCO and lowest unoccupied crystalline orbit (LUCO) positions. All MOF materials were characterized by an optical gap between 2.58 and 2.85 eV, making them excitable by visible light (λ > 420 nm). Notably, among the obtained MOF materials, ZrHf/BDC-NH_2_ and Zr@Hf/BDC-NH_2_ exhibited the highest negative LUCO potentials of −0.93 and −0.85 V, respectively, while Zr/BDC-NH_2_ and Hf/BDC-NH_2_ exhibited the most positive HOCO potentials of 2.75 and 2.66 V, respectively.

### 3.2 Photocatalytic Properties

#### 3.2.1 MMA Photopolymerization

Photopolymerization was performed using the obtained MOF materials, dissolved MMA, and ethyl-α-bromophenylacetate in DMF. As the co-initiator, ethyl-α-bromophenylacetate played an important role by participating in the generation of reactive radicals, which initiated MMA polymerization after excitation of the photochemical system. Analysis of PMMA production revealed that only ZrHf/BDC-NH_2_ was capable of producing considerable PMMA, with a yield of 47.7 mg in 4 h (Figure S18). By contrast, Ti/BDC-NH_2_, Zr/BDC-NH_2_, and Hf/BDC-NH_2_ exhibited negligible photocatalytic activity, with PMMA yields of only 3.5, 9.95, and 8.95 mg, respectively. Meanwhile, self-polymerization of MMA without a photocatalyst yielded only 4.65 mg of PMMA in 4 h. Notably, TiZr/BDC-NH_2_, TiHf/BDC-NH_2_, and TiZrHf/BDC-NH_2_ exhibited no photocatalytic activity while inhibiting the self-polymerization of MMA. Under the same conditions, the postsynthetically modified material, Zr@Hf/BDC-NH_2_, produced 28.7 mg of PMMA (Figure S19). The high photocatalytic activity of ZrHf/BDC-NH2 was attributed to its high radiation absorption efficiency, verified by its measured photocurrent (Figure S14) and highest LUCO level potential (Figure S17). MMA photopolymerization using ZrHf/BDC-NH_2_ for 2, 4, 8, 14, 24, and 44 h yielded 26.7, 47.7, 137.7, 286.2, 490.7, and 770.5 mg of PMMA, respectively (Figure S20).

A correlation analysis of selected properties (Figure S21) revealed that the horizontally oriented LUCO correlated negatively with pMAA production efficiency (−0.72), the presence of Ti correlated positively with larger particle size (+0.71) and higher absorbance at λ = 420 nm (+0.78), the BET value correlated positively with particle size (+0.76), and the optical gap correlated strongly and negatively with absorbance at λ = 420 nm.

Subsequently, photopolymerization efficiency using ZrHf/BDC-NH_2_ was investigated under different irradiation types (Figure 6a). Under light at λ > 400 nm, pMAA production increased to 182.7 mg in 4 h, while the reference process without a catalyst yielded 50 mg. By contrast, self-photopolymerization was more efficient under sunlight (AM 1.5 G) than using ZrHf/BDC-NH_2_ (267 vs 201 mg of pMAA). This discrepancy in production may have been due to the strong radiation energy of sunlight and light scattering during the reaction with the photocatalyst. Additionally, recycling ZrHf/BDC-NH_2_ three times revealed that the pMAA production efficiency slightly decreased from 48.1 mg in the first cycle to 40.9 mg in the third cycle (Figure 6b), which was attributed to loss of photocatalyst during recovery and cleaning. The stability of the material after MMA photopolymerization for 44 h was verified via SEM (Figure S22), TEM (Figure S23), and pXRD (Figure S24) analyses. Particle shape and size remained unchanged after 44 h (Figure S21), and the intensity and position of reflections were identical (Figure S24). Performing elemental mapping was not possible, likely because MMA molecules or small oligomers that formed during photopolymerization remained on the surface of the MOF material.

**Figure 6:**
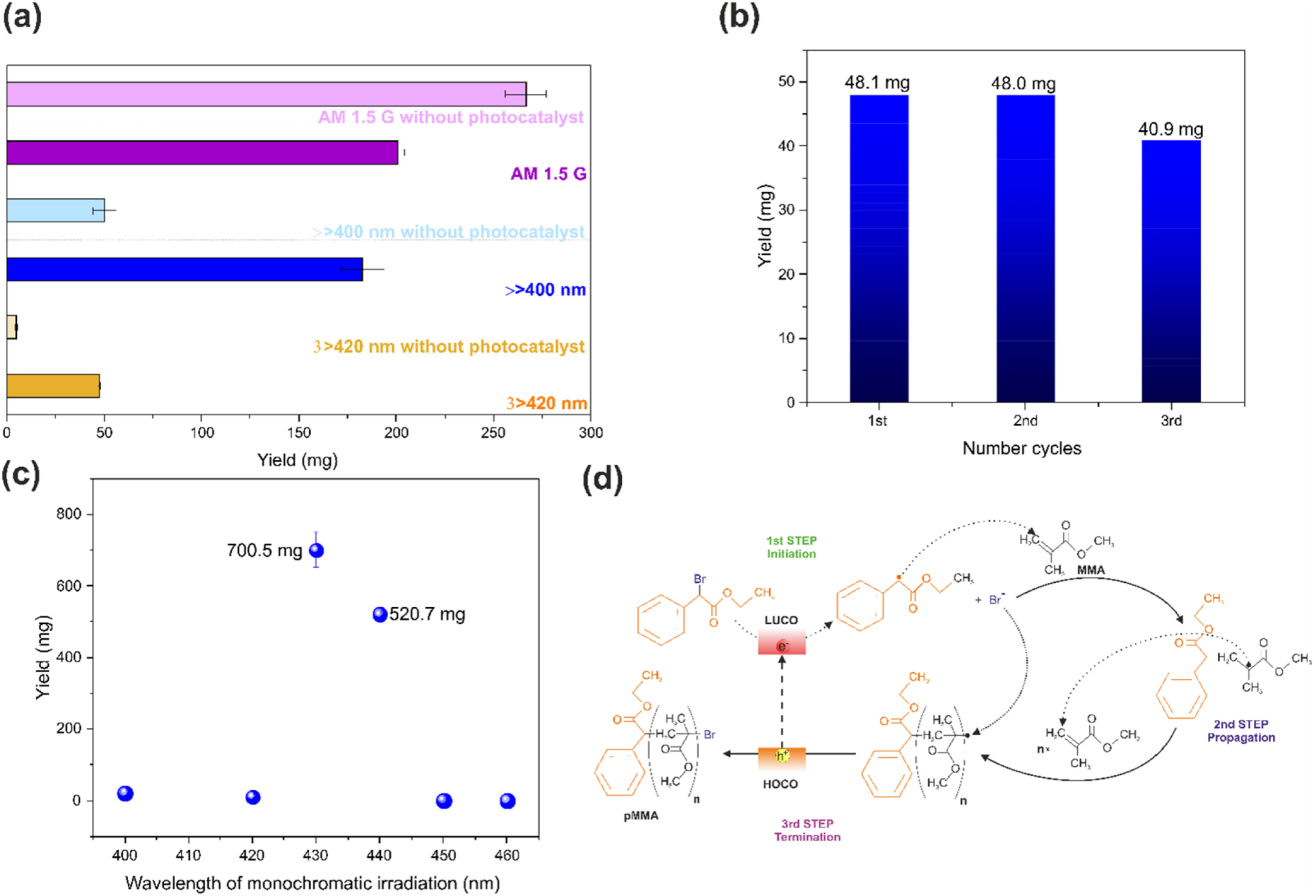
(a) Efficiency of MMA photopolymerization under different irradiation types (λ > 420 nm, λ > 400 nm, and sunlight [AM 1.5 G]) without or with ZrHf/BDC-NH_2_ (12 mg) for 4 h; (b) photostability of ZrHf/BDC-NH_2_ over 3 cycles under visible light (λ > 420 nm); (c) PMMA yield in 24 h under monochromatic radiation at different wavelengths with ZrHf/BDC-NH_2_ (12 mg); (d) proposed mechanism of photopolymerization used ZrHf/BDC-NH_2_.

Finally, the influence of the wavelength (λ) of monochromatic radiation (source: xenon lamp) on the efficiency of pMAA production using ZrHf/BDC-NH_2_ was investigated (Figure 6c). While monochromatic radiation at λ = 400, 420, 450, and 460 nm induced low photocatalytic activity (1.1–22.3 mg of pMAA in 24 h), that at λ = 430 and 440 nm induced the highest photocatalytic activity (700.5 and 520.7 mg of pMAA in 24 h, respectively). By contrast, MMA polymerization under monochromatic radiation at λ = 430 nm yielded 507 mg of pMAA in 44 h, which suggested degradation or dissolution of the polymer in its monomer. Notably, under monochromatic radiation at λ = 430 nm, pMAA production without a photocatalyst was only 4.2 mg in 24 h and 36.8 mg in 44 h. Furthermore, photopolymerization under LED radiation at λ = 430 nm yielded 87.5 mg of pMAA in 4 h (Figure S25).

#### 3.2.2 Characterization of PMMA

SEC analysis (Figures S26–S28) revealed high *M*_w_ values for all PMMA samples, ranging from 48 kDa (sunlight–AM 1.5 G) to 135 kDa (24 h, visible light at λ > 420 nm). Similar *M*_w_ values were obtained for PMMA samples obtained under visible light at λ > 420 nm for 4 and 14 h, indicating a response unrelated to reaction time. This phenomenon was attributed to the occurrence of a secondary process, such as continued depolymerization or chain-transfer reactions, which prevented an increase in polymer chain length. PDI (*M*_w_/*M*_n_) analysis revealed a relatively small distribution of values, ranging from 1.54 (2 h, visible light at λ > 420 nm) to 2.09 (44 h, visible light at λ = 430 nm). This finding suggested that the PMMA samples possessed slightly broad MWDs, which were beneficial for obtaining uniform physical and mechanical properties. Moreover, the *M*_w_ values of PMMA samples synthesized for 4 h under visible light at λ > 420 nm in the second and third cycles and under visible light at λ > 400 nm were comparable with those of samples synthesized for 24 h under visible light at λ > 420 nm, further confirming that prolonged reaction time did not lead to significantly longer polymer chain lengths. Instead, under the applied reaction conditions, an equilibrium occurred between chain growth and potential degradation processes (i.e., depolymerization). The PMMA samples displayed fairly high *M*_w_ values, with the highest recorded after 24 h (*M*_n_ = 72.9 kDa, *M*_w_ = 134.9 kDa). Additionally, of interest for statistical MWDs, the *M*_w_ values did not increase in direct proportion with reaction time: although the maximum *M*_w_ value was reached after irradiation for 24 h, longer polymerization (44 h) yielded lower *M*_w_ values, likely due to the occurrence of competitive depolymerization-type or chain-transfer reactions. The PDI values were typically in the 1.8–2.0 range, which is characteristic of free-radical polymerization. The PDI values were lowest for PMMA samples obtained under visible light at λ > 420 nm for 2 h and under LED radiation (1.54 and 1.64, respectively), indicating that, as expected, PMMA samples prepared via brief polymerization under LED conditions had narrower MWDs. By contrast, the PMMA sample synthesized under visible light at λ = 430 nm for 44 h exhibited the highest PDI value (2.09), confirming that protracted polymerization under modified conditions led to less uniform material. Compared to standard polymerization for 4 h, slight changes in reaction conditions (4 h, visible light at λ > 420 nm, second cycle, third cycle, and visible light at λ > 400 nm) increased the efficiency of PMMA production and yielded higher *M*_w_ values. Conversely, the PMMA sample obtained under UV–Vis light (AM 1.5 G) for 4 h exhibited a distinctly lower *M*_w_ value and higher PDI value (2.01), indicating inefficient chain growth. Similarly, the *M*_w_ values of PMMA samples prepared under λ = 430 nm conditions were markedly lower than those of their standard counterparts, indicating MWD broadening with reaction time. Among reaction times, results indicated that 24 h was ideal for MME photopolymerization. Although the longest reaction time yielded the highest *M*_w_ value, certain modified conditions led to chain degradation, resulting in lower *M*_w_ values and broader MWDs.

The FTIR spectra of PMMA samples (Figure S29) exhibited a broad spectral band from 3434 to 3651 cm^–1^, which was attributed to O–H stretching vibrations of hydroxyl groups, suggesting that water vapor was present on the surface or partial oxidation generated −OH functionalities. However, distinct bands at approximately 2999 and 2951 cm^–1^ were associated with the aliphatic C–H stretching vibrations of −CH_3_ and −CH_2_ groups present in the polymer main chain, respectively. A strong sharp band at 1732 cm^–1^, assigned to C=O ester stretching vibration, confirmed the presence of methacrylate ester functionalities. The band at 1625 cm^–1^ indicated potential C=C stretching vibrations, suggesting the presence of some unsaturated groups. Absorption at 1451 cm^–1^ was attributed to CH_3_ deformation vibrations. An additional absorption band at 1238 cm^–1^, which appeared to involve ν­(C–C–O) and ν­(C–O) stretching vibrations, further confirmed that the PMMA structure contained a major ester function. The band at 1154 cm^–1^ was ascribed to C–O–C (ester group) stretching vibrations, further confirming the PMMA structure.^54,55^ Finally, bands at 840 and 745 cm^–1^ emerged from out-of-plane bending vibrations of C–H groups. Overall, the results were consistent with the polymeric structure of PMMA.

The MALDI-TOF MS spectra of PMMA samples revealed multiple signals attributed to various levels of polymerization, with each molecule population having a constant part with a mass of ∼147 g/mol. Peaks at *m*/*z* 2700, 3449, 4303, 4824, 5386, 8202, 9759, 10,518, and 10,662 were assigned to monomer contributions, with the number of MMA units varying from approximately 25 to 105 (Figures S30–S35). Each mass difference for individual peaks represented a whole unit multiple of the monomer (100 g/mol), which verified the identification. Differences between theoretical and experimental data were attributed to various cationization mechanisms (H^+^, Na^+^, and K^+^) or the existence of various types of polymer end groups. The highest correlation was observed for H^+^ adducts for all samples and K^+^ adducts for peaks obtained for MMA8h, MMA14h, and MMA44h. The results were consistent with a fraction containing two high-*M*_w_ polymers, and analysis of the entire MALDI-TOF-MS spectrum indicated the presence of oligomers with varying chain lengths.

The ^1^H NMR spectra confirmed the structure of PMMA (Figures S36–S38). The ^1^H NMR spectrum for PMMA obtained for 8 h under visible light at λ > 420 nm displayed characteristic signals: a strong singlet at δ = 3.60 ppm corresponding to the OCH_3_ protons of the methyl group, a broad signal at δ = 1.81 ppm assigned to the CH and CH_2_ protons of the polymer backbone, and signals in the δ = 0.85–1.02 ppm region attributed to the protons of side methyl groups. Further less-intense resonances in the δ = 2.8–3.0 ppm region were assigned to the benzylic protons of the chain end introduced through the co-initiator, ethyl-α-bromophenylacetate. A residual CHCl_3_ signal (δ = 7.26 ppm) was attributed to the solvent and tetramethylsilane reference. The resulting spectrum aligned with the structure of PMMA obtained via radical polymerization, indicating its repetitive structure and initiator end group at low concentration.

#### 3.2.3 Mechanism of Photopolymerization

A mechanism for the excitation and photopolymerization of MMA was proposed based on the available literature and experiments with scavengers, as illustrated in Figure 6d.^56,57^ MMA photopolymerization with ZrHf/BDC-NH_2_ was performed under visible light using DMSO to scavenge electrons and EDTA to scavenge holes (Figure S39). On the one hand, the presence of DMSO in the system suppressed PMMA production, which was attributed to the substantial role of electrons in MMA photopolymerization. On the other hand, the presence of EDTA increased the PMMA production yield to 320 mg (with more fragment ions of higher mass, as shown in Figure S40), which was attributed to the inhibition of polymerization termination by holes and the formation of a high-*M*_w_ polymers. Results suggested that the excited photocatalyst (ZrHf/BDC-NH_2_) generates a pair of reactive forms: *e* ^*–*^ at the LUCO level and *h* ^*+*^ at the HOCO level. In the first stage, the co-initiator (ethyl-α-bromophenylacetate) is reduced to form a phenyl acetate radical and detach the bromide anion. The reactive radical then attacks MMA, forming a radical compound that can attach subsequent monomers in succession during the propagation stage. In the termination stage, the bromide ion attaches to a long-chain radical, which is oxidized to form PMMA with attached co-initiator elements.

## 4 Conclusions

Hot injection was successfully applied for obtain NH_2_-UiO-66 particles with mono (Zr or Hf) and bimetallic (Zr and Hf) metal centers. The resulting MOF materials exhibited high crystallinity and porosity, comparable to those obtained via conventional methods. Furthermore, the hot injection method applied to particles containing Ti, Zr, and Hf centers yielded MOF materials with moderate crystallinity, whereas combinations of Ti–Zr and Ti–Hf resulted in amorphous materials. In addition, ZrHf/BDC-NH_2_ demonstrated high photocatalytic activity for MMA photopolymerization, exhibiting particularly high efficiency under monochromatic radiation at λ = 430 nm (xenon lamp or LED source). Results suggest that the proposed method is a promising approach for scaling up and optimizing the synthesis of other MOFs. Notably, MME photopolymerization using ZrHf/BDC-NH_2_ represents a viable alternative to conventional methods, and the use of inexpensive LED irradiation to induce photopolymerization highlights the advantage of the proposed method for future practical applications.

## Supplementary Material



## Data Availability

The data supporting the findings of this study are openly available in Repository for Open Data—RepOD at 10.18150/OBXDPQ. The data supporting this study have been included as part of the Supporting Information.

## Notes

Additional experimental details and characterization data for all samples, including synthesis parameters, structural and sorption properties of MOFs, surface and microscopic analyses, diffraction, spectroscopic and thermal measurements, photocatalytic MMA polymerization studies, and molecular weight and spectroscopic characterization of PMMA (PDF)

## Notes

M.A.B.: Project administration, Conceptualization, Formal analysis, Data curation, Methodology, Resources, Investigation, Validation, Funding acquisition, Visualization, Writing—original draft, Writing—review and editing. M.M.-M.: Investigation; Visualization; Conceptualization; Methodology; Writing—original draft; Writing—review and editing. J.D.: Investigation; Visualization, Methodology; Writing—original draft. K.N.: Investigation, Writing—original draft. K.M.: Investigation. T.K.: Investigation. S.Z.: Investigation; Visualization. A.Z.-M.: Supervision, Writing—review and editing
