# Supplementary material for: Rapid Synthesis of MIV-Based Multimetal–Organic Frameworks for Photopolymerization of Methyl Methacrylate under Visible Light
Source: ACS Appl Mater Interfaces. 2026 Mar 16;18(12):17895–904. doi: 10.1021/acsami.6c00818 (PMC13318065; doi:10.1021/acsami.6c00818)
Supplement: Supplementary file 1 [file am6c00818_si_001.pdf]

Supporting Information

Rapid synthesis of M^{IV}-based multimetal–organic frameworks for photopolymerization of methyl methacrylate under visible light

Mateusz Adam Baluk[1], Magdalena Miodyńska-Melzer[1*], Joanna Drzeżdżon[1], Kostiantyn Nikiforow[2], Tomasz Klimczuk[3,4], Krzysztof Matus[5], Stefania Zappia[6], Adriana Zaleska-Medynska[1**]

*magdalena.miodynska-melzer@ug.edu.pl

** adriana.zaleska-medynska@ug.edu.pl

[1] Department of Environmental Technology, Faculty of Chemistry, University of Gdańsk, Wita Stwosza 63, 80-308 Gdansk, Poland

[2] Institute of Physical Chemistry, Polish Academy of Science, Kasprzaka 44/52, 01-224 Warsaw, Poland

[3] Faculty of Applied Physics and Mathematics, Gdansk University of Technology, Narutowicza 11/12, 80-233 Gdansk, Poland

[4] Advanced Materials Center, Gdansk University of Technology, Gdansk, Poland

[5] Silesian University of Technology, Gliwice, Poland

[6] Istituto di Scienze e Tecnologie Chimiche “Giulio Natta” (SCITEC) of the Consiglio Nazionale delle Ricerche (National Research Council - CNR), via Alfonso Corti 12, 20133 Milano, Italy

Table S1. Comparison of conditions for indirect free radical photopolymerization using different photocatalysts.

Photocatalyst	Type of irradiation	Co-initiator	Photo-catalyst amount (mg)	MMA amount (mL)	Time (h)	Yield	Mn (kDa)	PDI	Ref.
ZrHf/BDC-NH_2	Vis > 420 nm	EBPA	12	4	4	48.1 mg	62.7	1.90	This work
ZrHf/BDC-NH_2	LED 430 nm	EBPA	12	4	4	87.5 mg	32.2	1.64	This work
MOF-901 (Ti)	Vis (fluor.)	EBPA	10	2	40	60%	29	1.60	1
ZnO (QDs)*	UV (355 nm)	-	~5	5	0.5	~10%	40	2.10	2
CdS QDs	Vis (λ > 400 nm)	TEA	2.5	1	2	~45%	150	1.90	3
g-C_3N4	Vis (λ > 420 nm)	TEA	20	5	24	38%	12.5	1.85	4
NNU-28 (Zr)	Vis (λ > 420 nm)	TEA	5	1	12	81%	11.2	1.25	5
TiO_2 (NPs)*	UV	Carboxylic acids	10	2	2-6	~5 mg	25-50	>2.0	6

(EBPA – ethyl α-bromophenylacetate; TEA – triethylamine).

Table S2. Synthesis parameters and crystal structures of the materials.

Name	Amount of $Ti(OPr)_4$	Amount of $Zr(OPr)_4$	Amount of $Hf(OBut)_4$	Crystal structure	Size (nm)	Obtained shape	BET surface area (m^2/g)	CO_2 uptake at 1 bar (mmol/g)
Ti/BDC-NH_2	4 mmol	-	-	NH_2-MIL-125 (Ti)	579 ± 137	octahedra particles	646	1.98
Zr/BDC-NH_2	-	4 mmol	-	NH_2-UiO-66 (Zr)	51 ± 15	similar to octahedra particles	473	1.30
Hf/BDC-NH_2	-	-	4 mmol	NH_2-UiO-66 (Hf)	19 ± 9	mostly irregular, some similar to octahedra	278	0.60
TiZr/BDC-NH_2	2 mmol	2 mmol	-	amorphous	29 ± 16	mostly irregular, some similar to octahedra	274	0.56
TiHf/BDC-NH_2	2 mmol	-	2 mmol	amorphous	19 ± 9	irregular small particles	151	0.39
ZrHf/BDC-NH_2	-	2 mmol	2 mmol	NH_2-UiO-66 (Zr/Hf)	43 ± 15	similar to octahedra	392	0.91
TiZrHf/BDC-NH_2	1.3 mmol	1.3 mmol	1.3 mmol	amorphous	40 ± 18	irregular small particles	278	0.51
Zr@Hf/BDC-NH_2	-	4.3 mmol*	4 mmol	NH_2-UiO-66 (Zr/Hf)	27 ± 15	unchanged shape as Hf/BDC-NH_2	76	0.28

*4 mmol of $ZrCl_4$ as postsynthetical modification

Table S3. XPS study results of samples.

	Ti/BDC-NH_2		Zr/BDC-NH_2		Hf/BDC-NH_2		TiHf/BDC-NH_2		TiZr/BDC-NH_2		ZrHf/BDC-NH_2		TiZrHf/BDC-NH_2		Zr@Hf/BDC-NH_2	
	BE (eV)	At. (%)	BE (eV)	At. (%)	BE (eV)	At. (%)	BE (eV)	At. (%)	BE (eV)	At. (%)	BE (eV)	At. (%)	BE (eV)	At. (%)	BE (eV)	At. (%)
Me-O	529.72	**4.72**	530.29	**4.06**	530.15	**4.58**	530.22	**9.89**	530.59	**14.23**	529.99	**6.19**	529.04	**1.62**	529.59	**6.92**
Me-O-C	530.83	**11.82**	531.67	**17.86**	531.24	**13.56**	531.55	**17.36**	532.08	**23.17**	531.29	**16.1**	530.66	**13.88**	531.07	**20.73**
C-O	532.07	**18.62**	532.56	**12.29**	532.37	**19.31**	532.79	**11.54**			532.51	**15.37**	532.1	**20.79**	532.57	**17.07**
O (sum.)		**35.16**		**34.21**		**37.45**		**38.79**		**37.4**		**37.66**		**36.29**		**44.72**
C-NH2	399.58	**5.53**	399.81	**6.91**	399.55	**5.35**	399.73	**5.96**	399.56	**4.93**	399.67	**5.88**	399.62	**6.33**	399.85	**4.04**
C-NH+	402.33	**1.51**							401.71	**1.24**						
N (sum.)		**7.04**		**6.91**		**5.35**		**5.96**		**6.17**		**5.88**		**6.33**		**4.04**
C-C	284.8	**32.12**	284.8	**30.42**	284.8	**29.83**	284.8	**26.86**	284.8	**26.95**	284.84	**28.08**	284.8	**32.28**	284.74	**18.32**
C-N\C-O	286.53	**6.83**	286.16	**8.79**	286.06	**6.4**	286.78	**8.44**	286.27	**7.96**	286.1	**7.28**	286.3	**3.54**	286.13	**8.3**
COOH	288.83	**11.84**	288.9	**13.26**	288.95	**13.17**	289.11	**11.8**	288.9	**13.21**	288.98	**13.4**	288.78	**13.69**	288.92	**12.64**
C (sum.)		**50.79**		**52.47**		**49.4**		**47.1**		**48.12**		**48.76**		**49.51**		**39.26**
Ti-O	458.93	**4.68**			-		459.08	**2.58**	459.09	**3**	-		458.93	**1.18**	-	
	464.64	**2.34**			-		464.87	**1.29**	464.82	**1.5**	**-**		464.63	**0.59**	-	
		**7.02**	-					**3.87**		**4,5**	**-**			**1,77**	-	
Hf-O			-		17.51	**4.46**	17.42	**2.45**	-		17.49	**2.79**	17.38	**1.79**	17.22	**1.96**
			-		19.14	**3.34**	19.12	**1.84**	-		19.07	**2.09**	19.08	**1.34**	18.81	**1.47**
						**7,8**		**4,29**	-			**4.88**		**3,13**		**3,43**
Zr-O	-		182.97	**3.86**			**-**		182.61	**1.49**	182.26	**0.96**	182.2	**0.78**	182.22	**3.7**
	-		185.35	**2.57**			**-**		185.01	**1**	184.66	**0.64**	184.6	**0.52**	184.62	**2.47**
	-		183.21	**2.62**			**-**		183.34	**0.78**	185.8	**0.49**	183.17	**0.99**	183.43	**1.36**
			185.61	**1.75**					185.74	**0.52**	185.9	**0.12**	185.57	**0.66**	185.83	**0.91**
				**6.41**						**3.79**		**2,82**		**2.95**		**8.44**

Table S4. Influence of irradiation type on the photocatalytic polymerization of methyl methacrylate using ZrHf/BDC-NH_2 as photocatalysts.

Conditions production of PMMA	reaction time (h)	Amount of mers for the longest chains	M_n (g mol^{-1})^a	M_w (g mol^{-1})^b	PDI Mw/Mn^c
4 h, Vis > 420 nm	4	52 and 105 mers	62763	119139	1.90
44 h, Vis > 420 nm	44	33 and 52 mers	54478	107560	1.97
24 h, Vis > 420 nm	24	33 mers	72910	134890	1.85
14 h, Vis > 420 nm	14	33 and 41 mers	66135	126690	1.92
8 h, Vis > 420 nm	8	33, 52, and 80 mers	52336	94203	1.80
2 h, Vis > 420 nm	2	33 and 47 mers	43153	66381	1.54
4 h, Vis > 420 nm, 2nd cycle	4	103 mers	66726	133550	2.00
4 h, Vis > 420 nm, 3rd cycle	4	33, 52, and 96 mers	67155	125935	1.88
4 h, Vis > 400 nm	4	33 and 52 mers	67198	122251	1.82
4 h, UV-Vis – AM 1.5 G	4	25, 33, and 52 mers	23693	47542	2.01
4 h, Vis = 430 nm LED	4	33, 41, and 52 mers	32237	52730	1.64
24 h, Vis = 430 nm	24	33 and 47 mers	35799	66110	1.85
44 h, Vis = 430 nm	44	33 and 52 mers	30253	63102	2.09

M_n = number-average molecular weight, ^{b} M_w = weight-average molecular weight,
^{c} PDI = Polydispersity index (Mw/Mn)

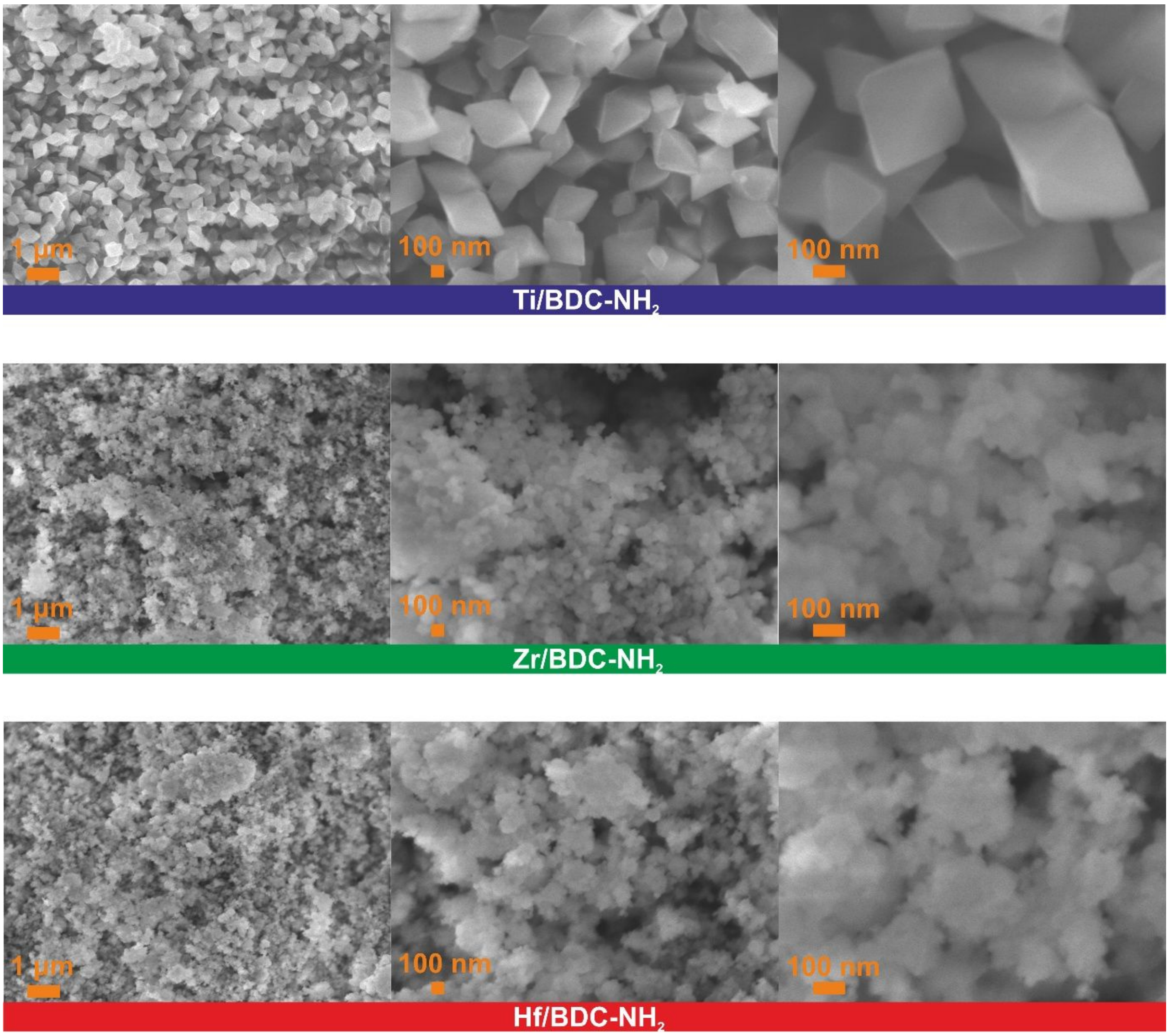

Figure S1. SEM images of Ti/BDC-NH_2, Zr/BDC-NH_2, Hf/BDC-NH_2.

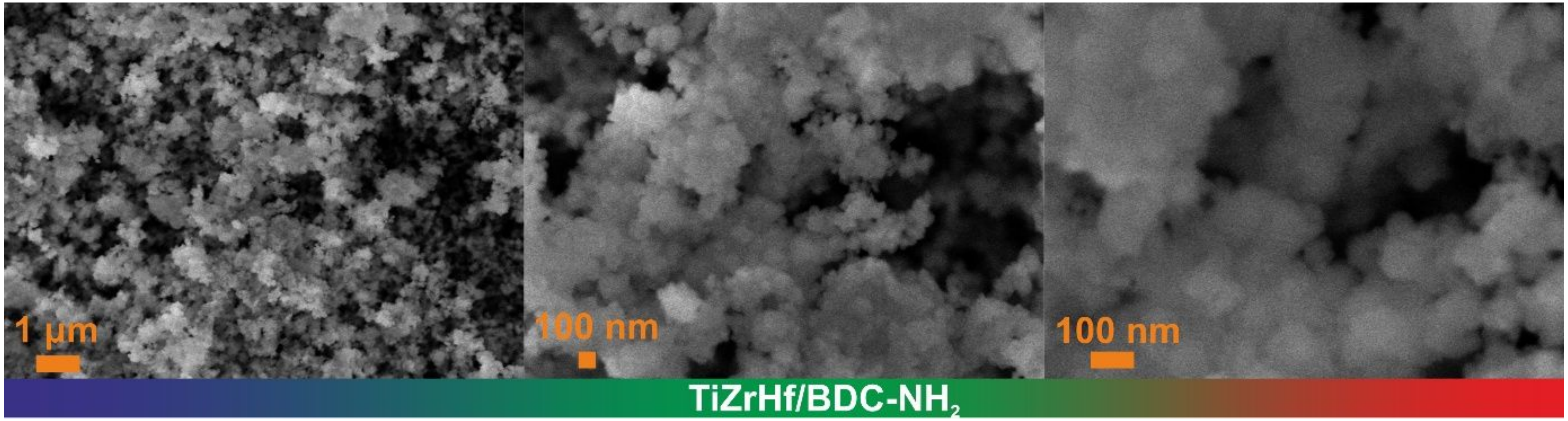

Figure S2. SEM images of TiZrHf/BDC-NH_2.

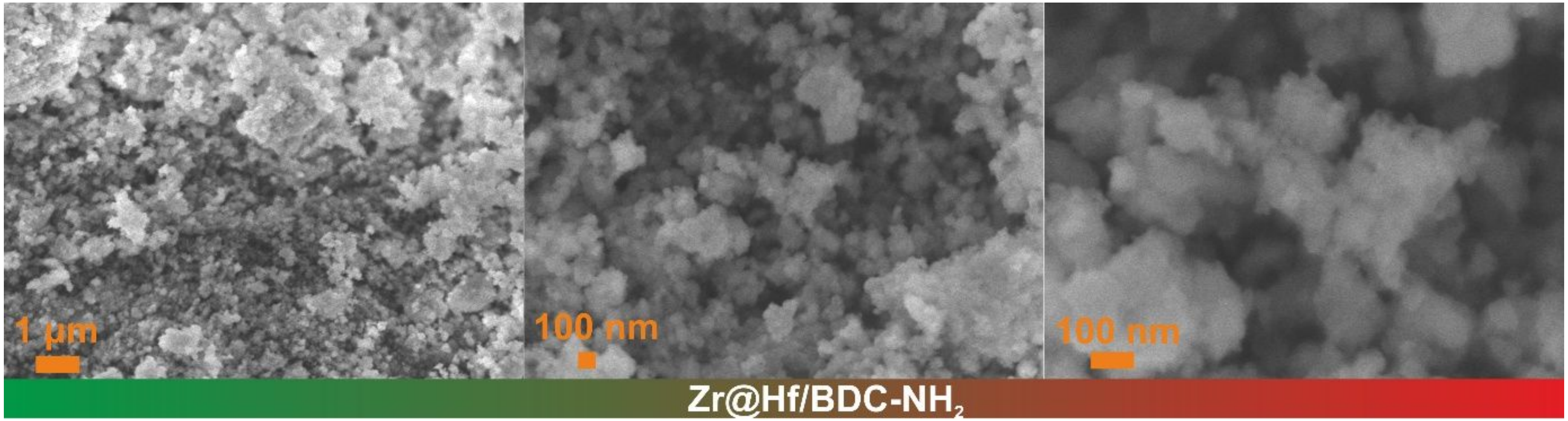

Figure S3. SEM images of Zr@Hf/BDC-NH_2.

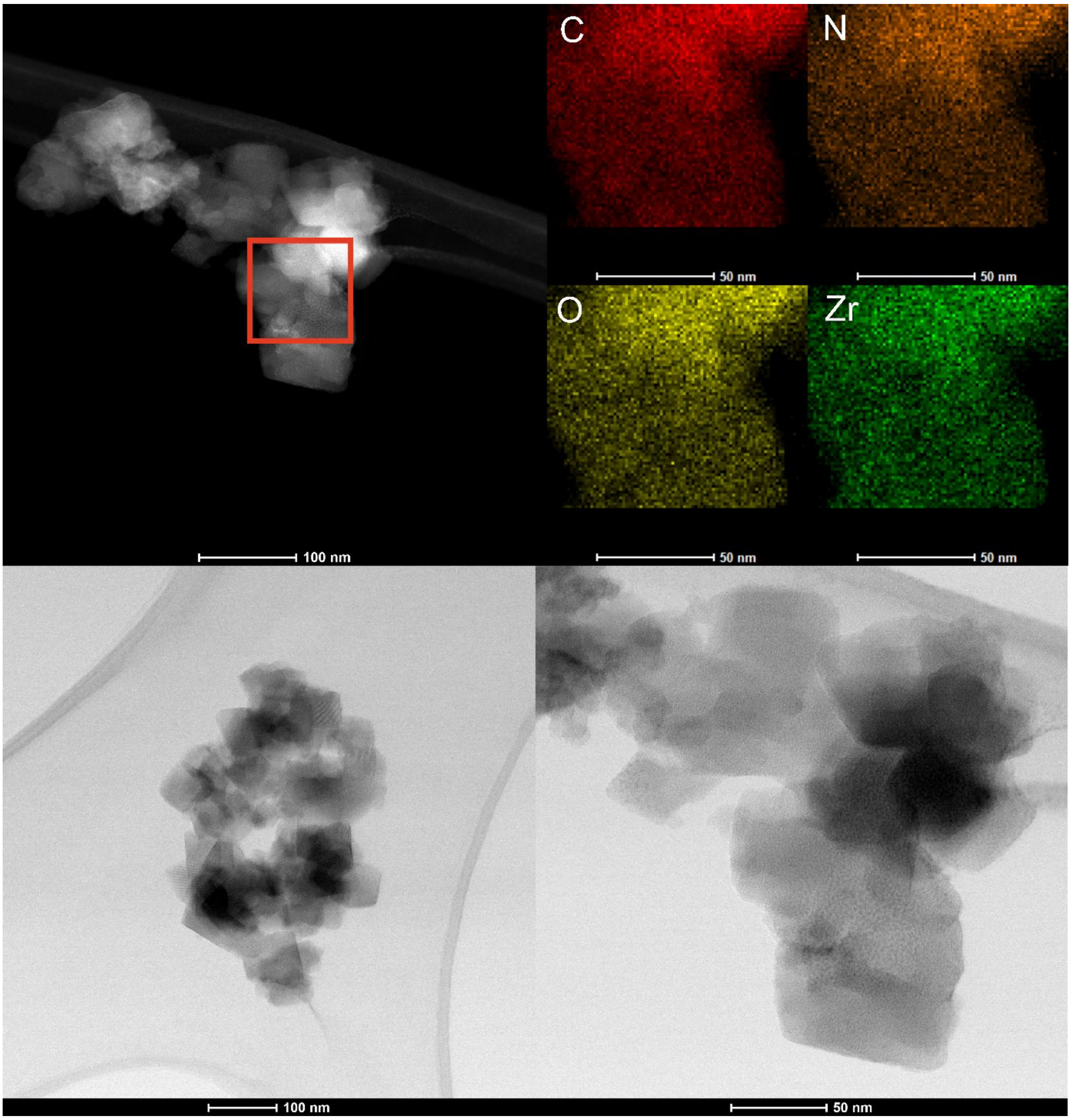

Figure S4 TEM and STEM images with EDS mapping (C, N, O and Zr) for Zr/BDC-NH_2

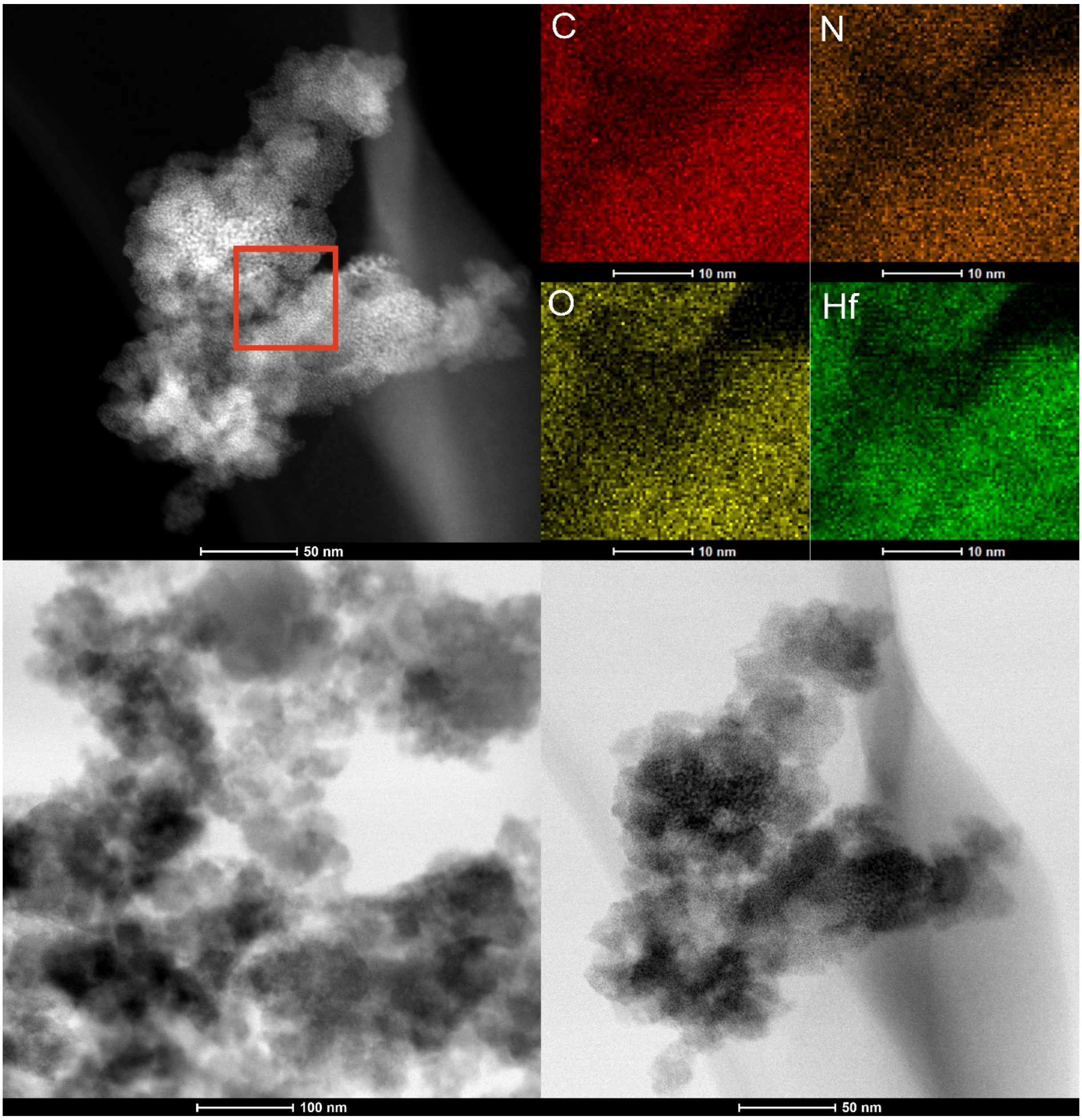

Figure S5. TEM and STEM images with EDS mapping (C, N, O and Hf) for Hf/BDC-NH_2

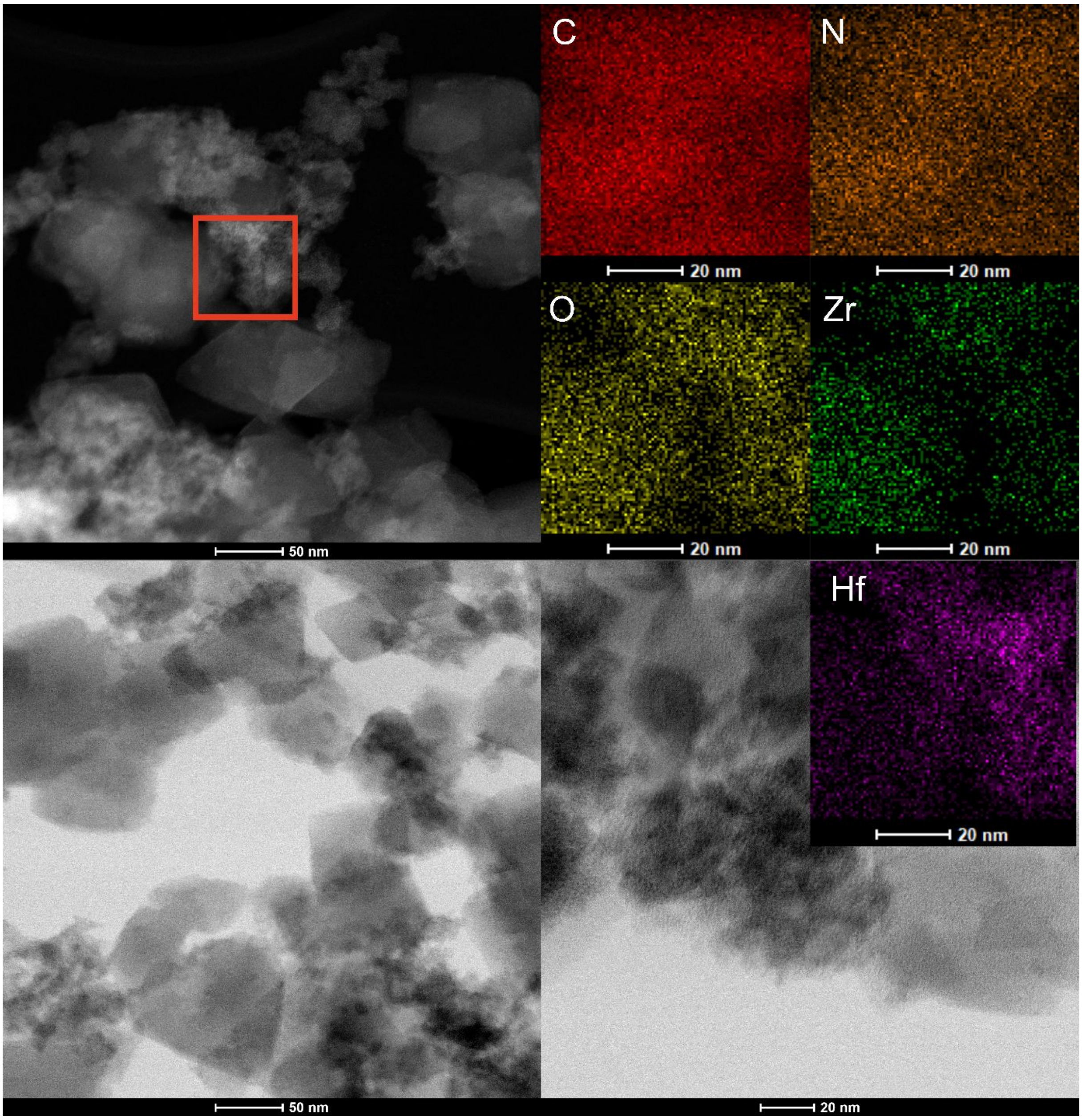

Figure S6. TEM and STEM images with EDS mapping (C, N, O, Zr and Hf) for ZrHf/BDC-NH_2

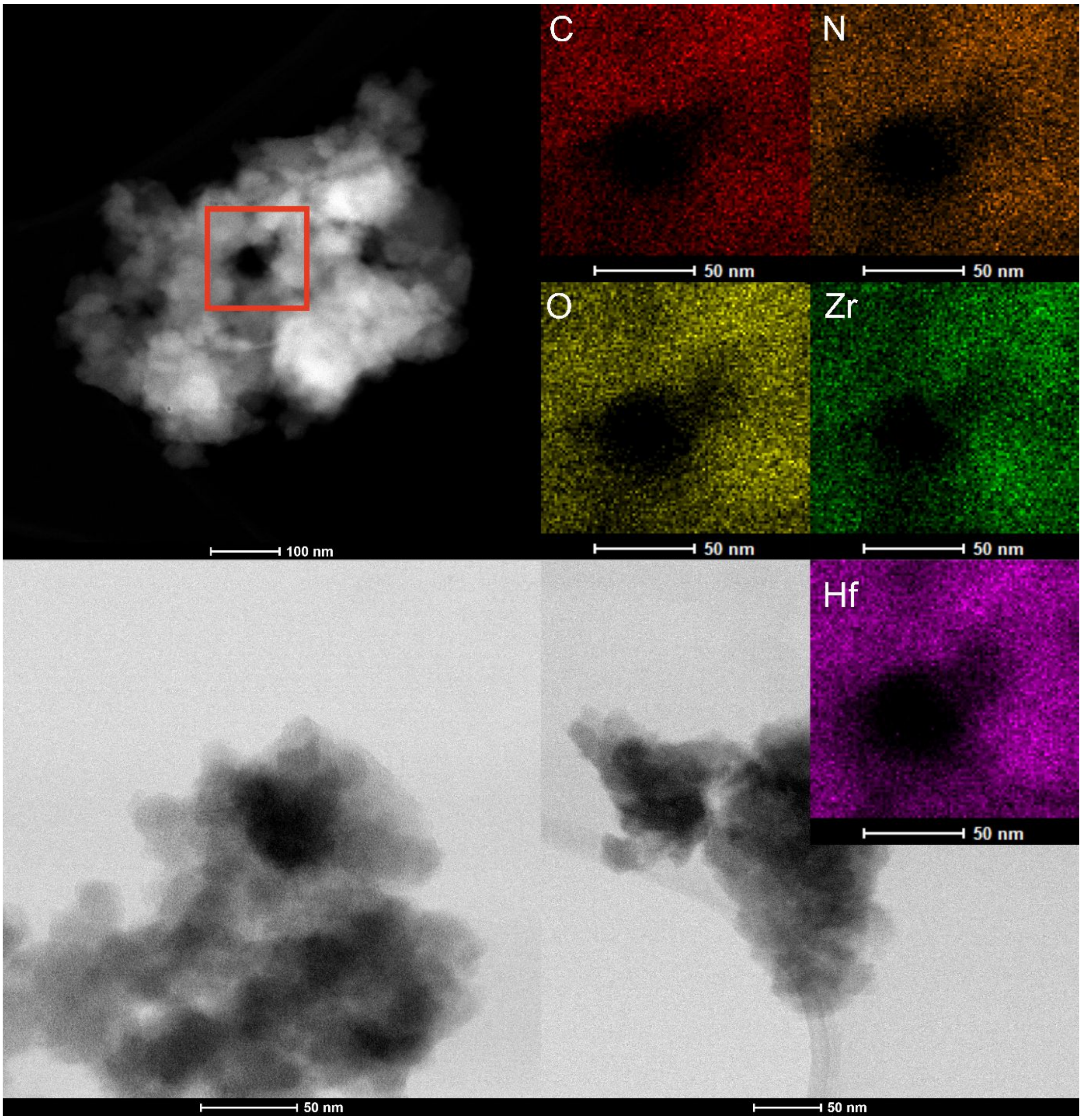

Figure S7. TEM and STEM images with EDS mapping (C, N, O, Zr and Hf) for Zr@Hf/BDC-NH_2

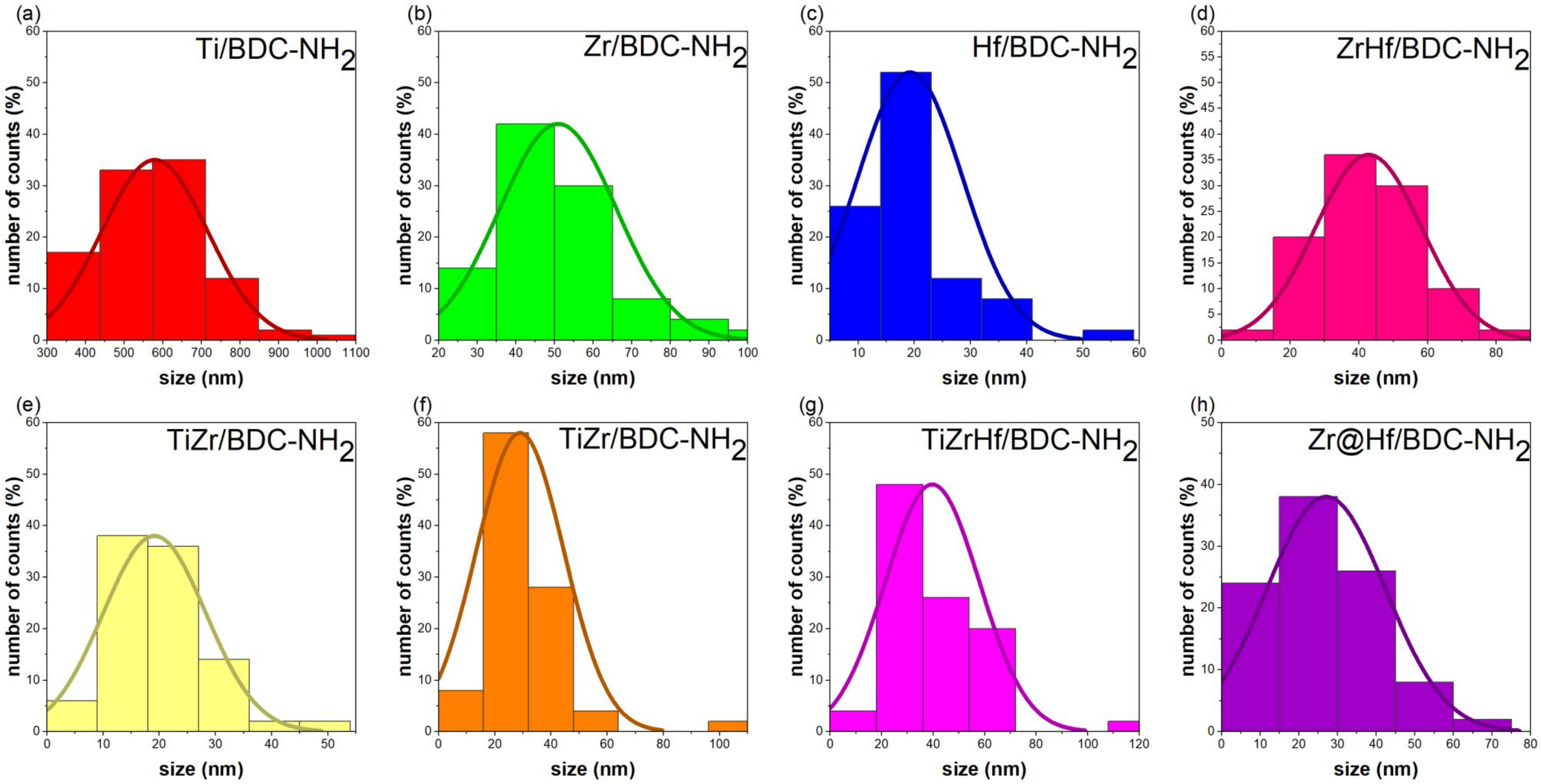

Figure S8. Particle size distribution of (a) Ti/BDC-NH_2, (b) Zr/BDC-NH_2, (c) Hf/BDC-NH_2, (d) ZrHf/BDC-NH_2, (e) TiZr/BDC-NH_2, (f) TiZr/BDC-NH_2, (g) TiZrHf/BDC-NH_2 and (h) Zr@Hf/BDC-NH_2 (counted based on 50 counted values, distribution as standard deviation)

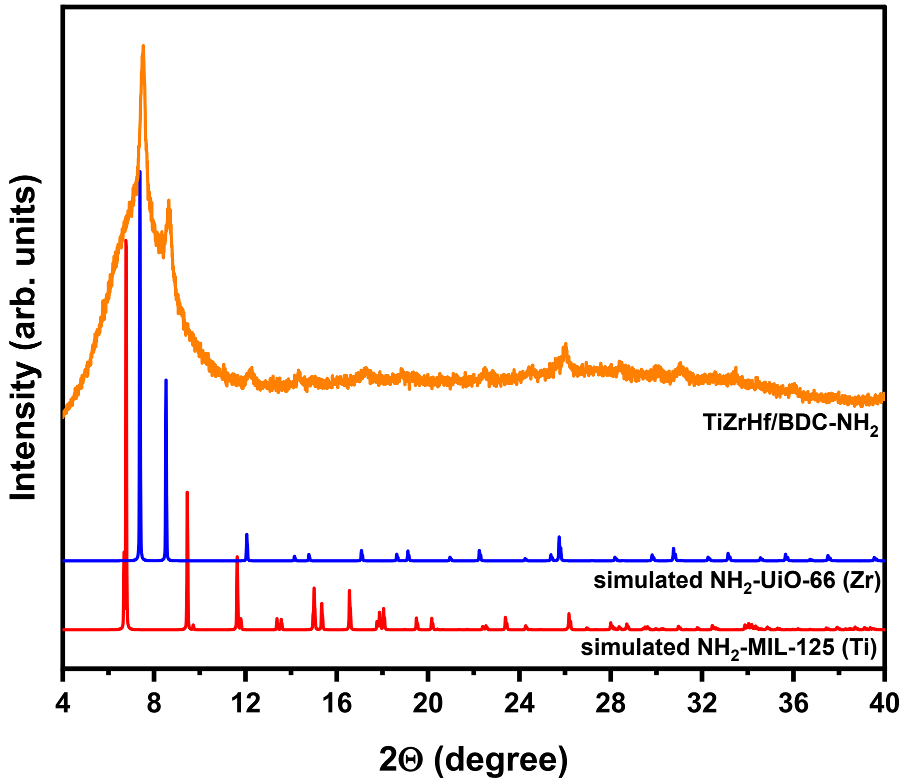

Figure S9. XRD analysis of TiZrHf/BDC-NH_2 with simulated plot from VESTA Software[7] (No. card 7211159 for NH_2-MIL-125 (Ti) and 4512072 for NH_2-UiO-66 (Zr) from Crystallography Open Database[8]).

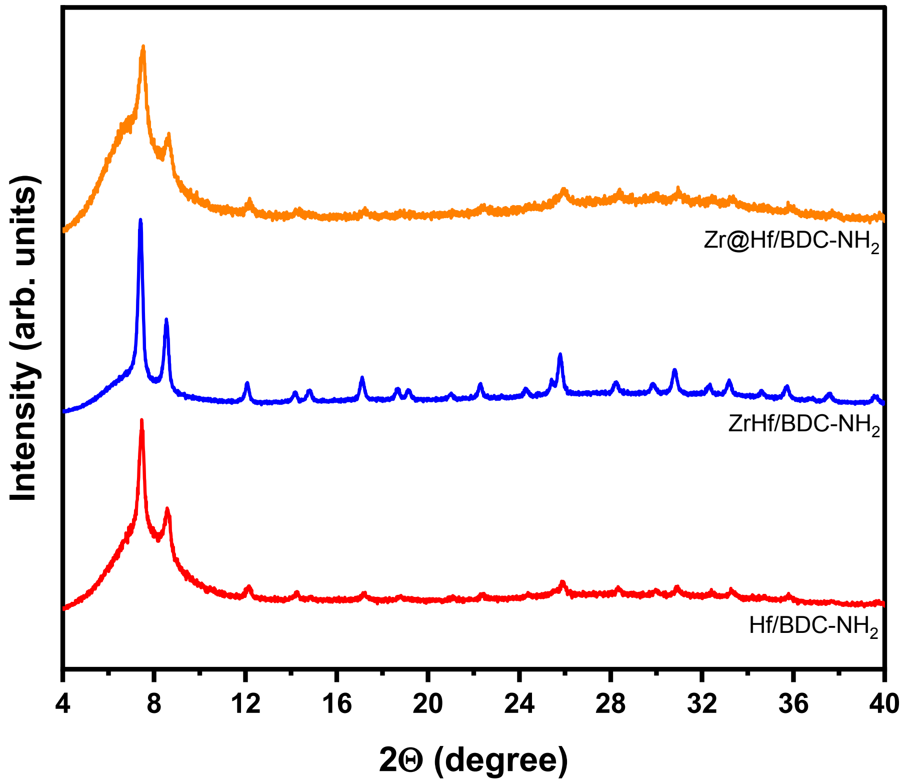

Figure S10. XRD analysis of Zr@Hf/BDC-NH_2 with ZrHf/BDC-NH_2 and Hf/BDC-NH_2.

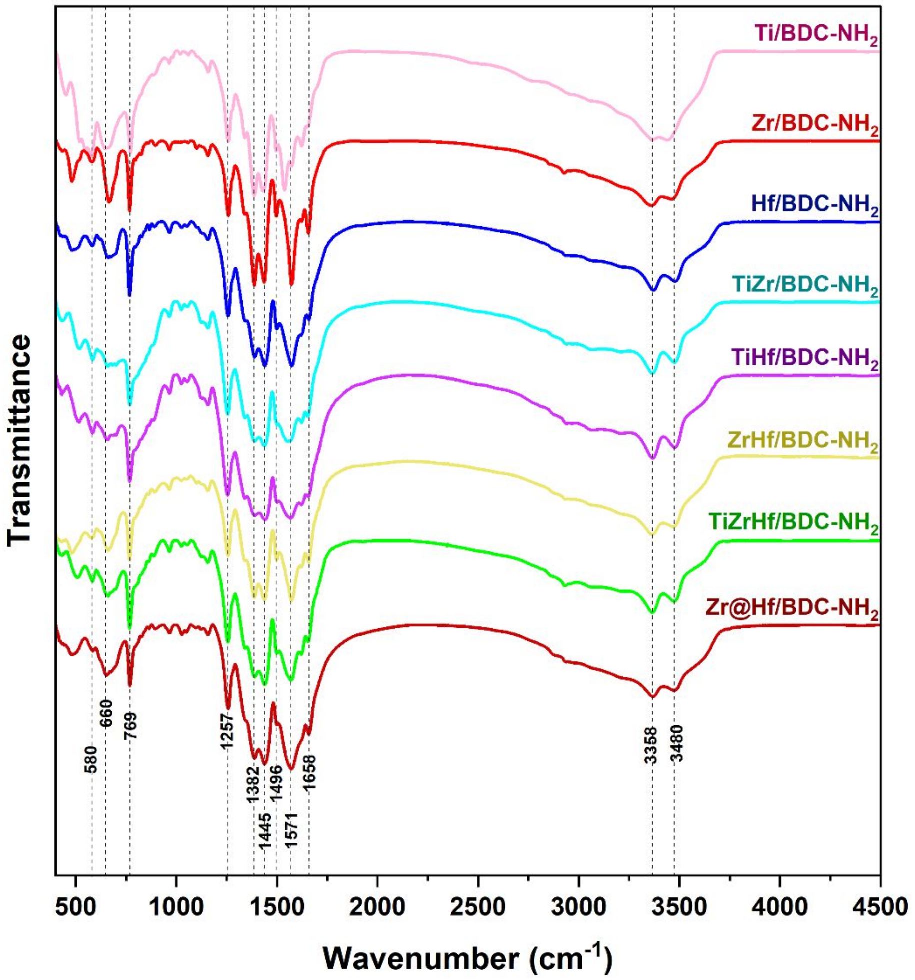

Figure S11. FTIR spectra of obtained materials.

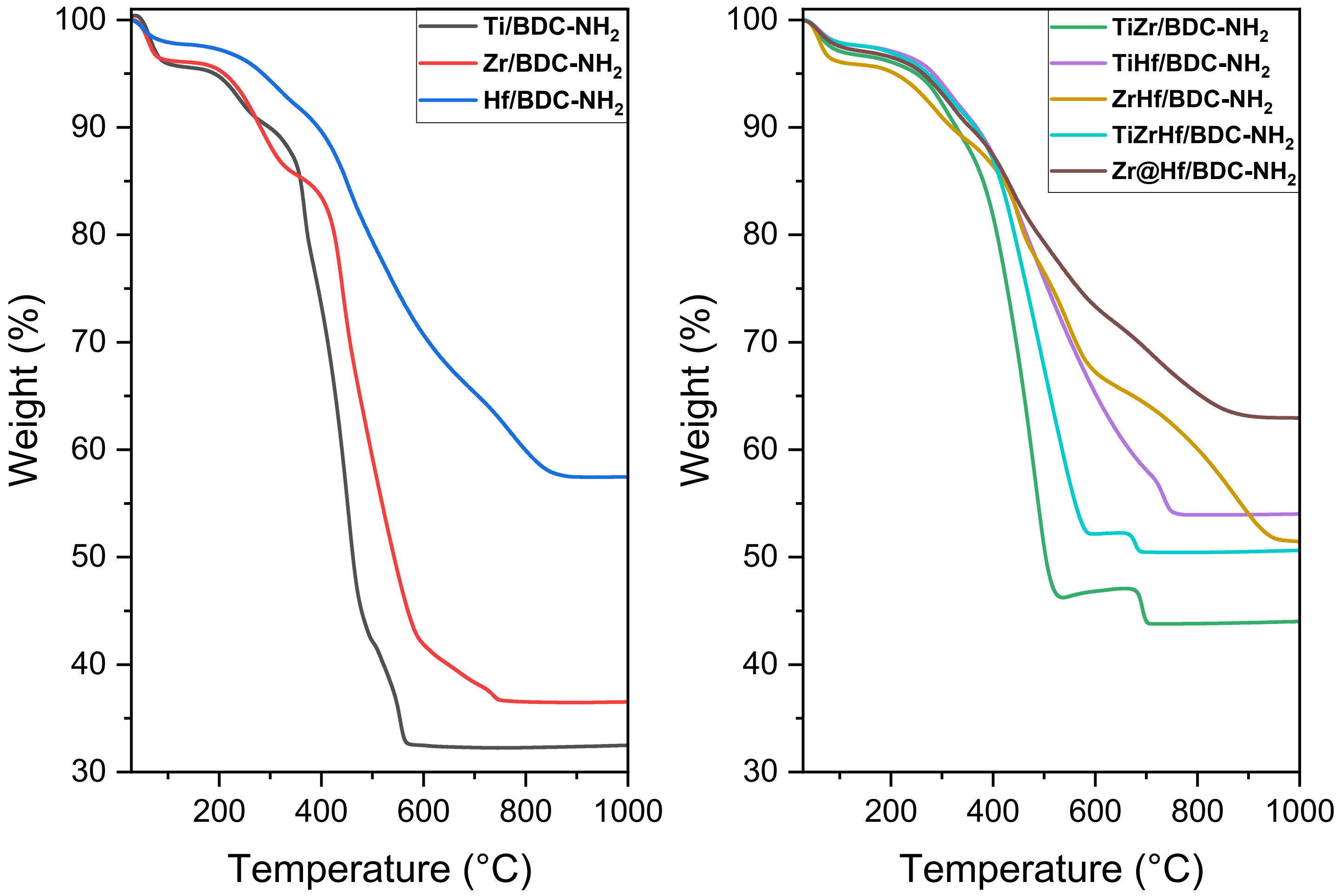

Figure S12. TG analysis of obtained materials.

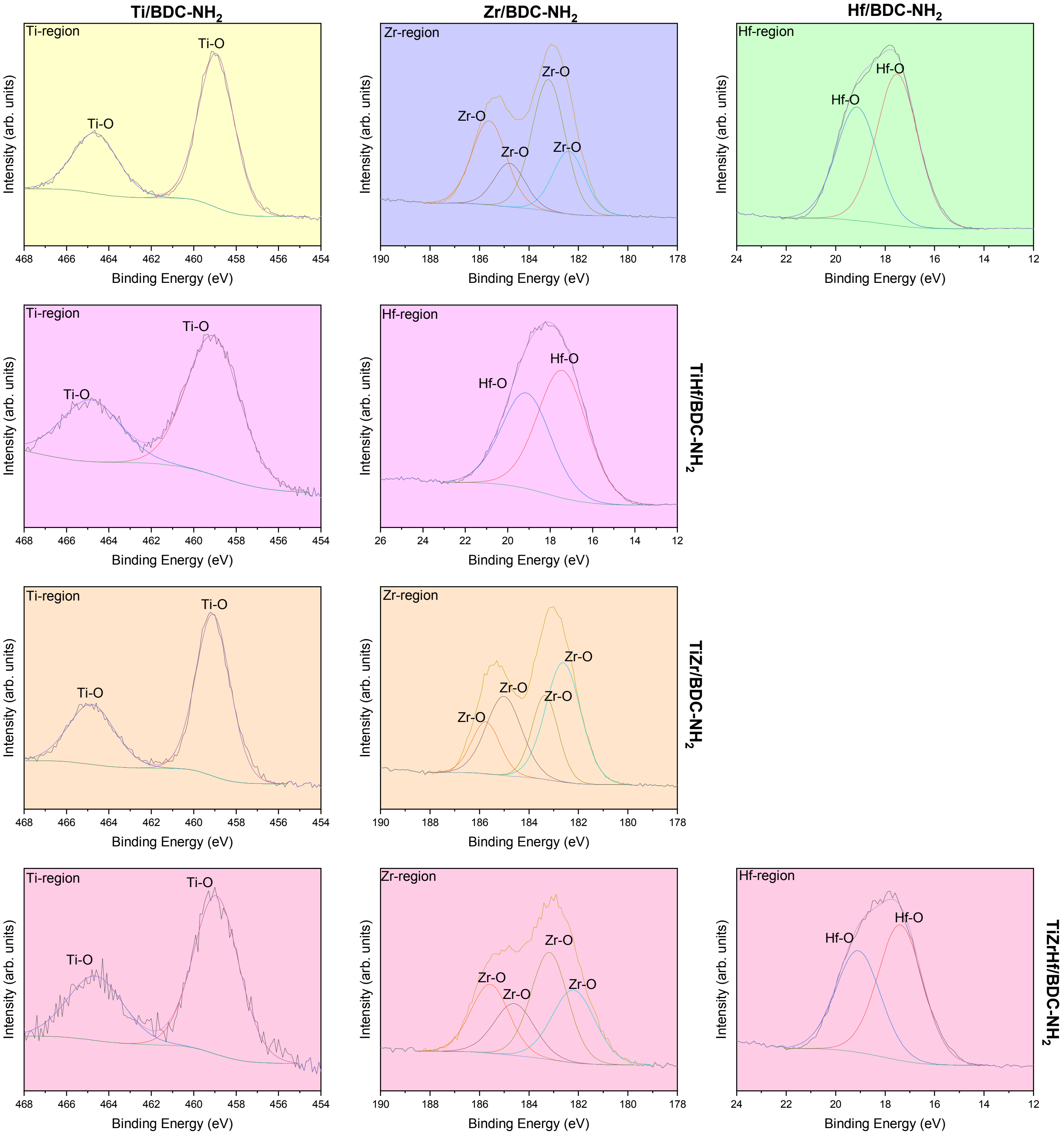

Figure S13. High resolution spectra of Ti, Zr or Hf for Ti/BDC-NH_2, Zr/BDC-NH_2, Hf/BDC-NH_2, TiZr/BDC-NH_2, TiHf/BDC-NH_2 and TiZrHf/BDC-NH_2.

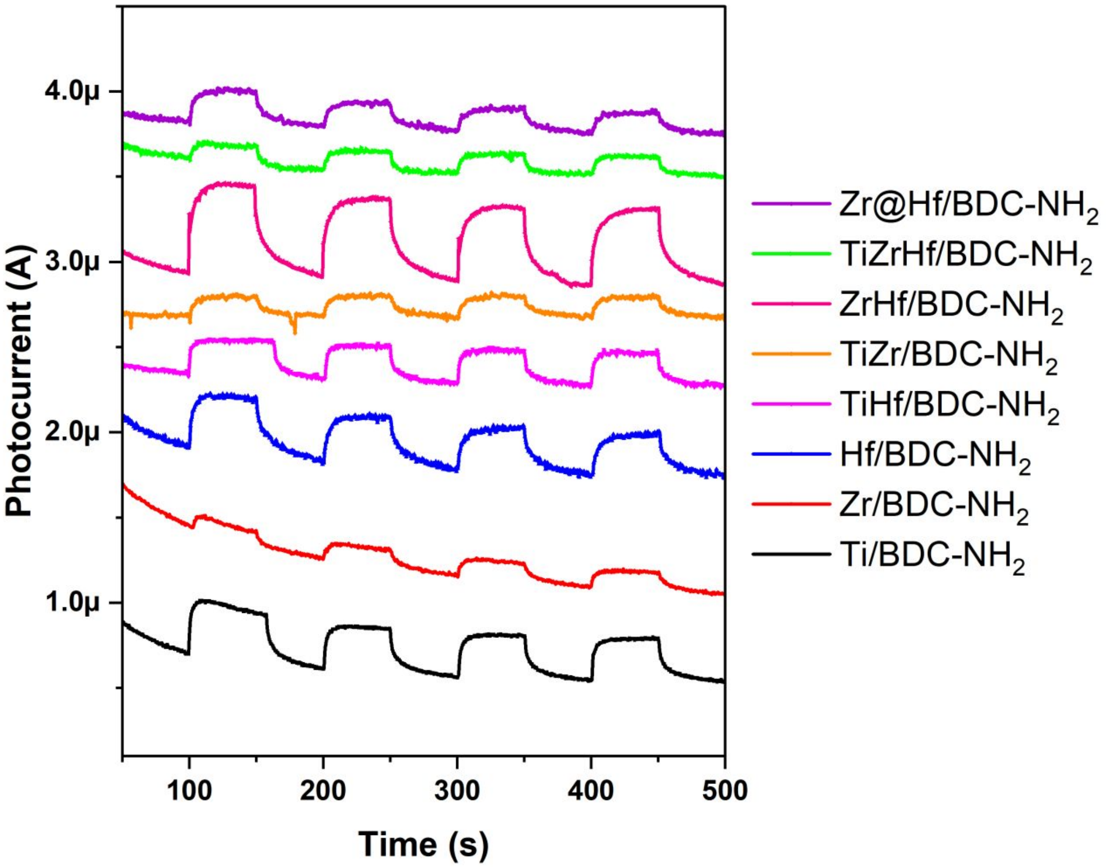

Figure S14. Transient photocurrent responses of obtained samples in 0.1 M Na_2SO_4 aqueous solution without bias versus Ag/AgCl upon UV-Vis light.

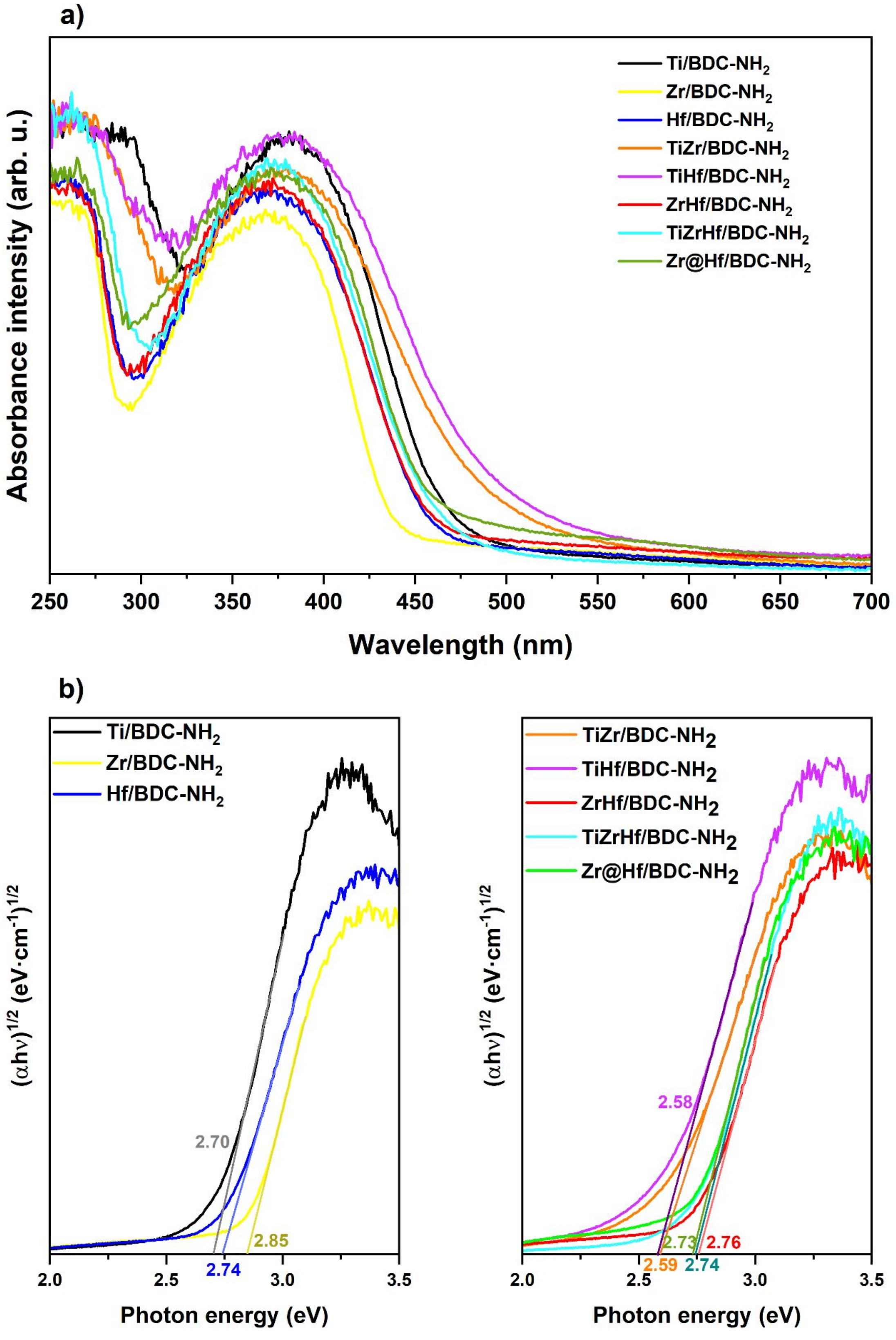

Figure S15. a) UV-Vis/DRS spectra of obtained materials; b) Tauc plots for the calculation of their optical gap.

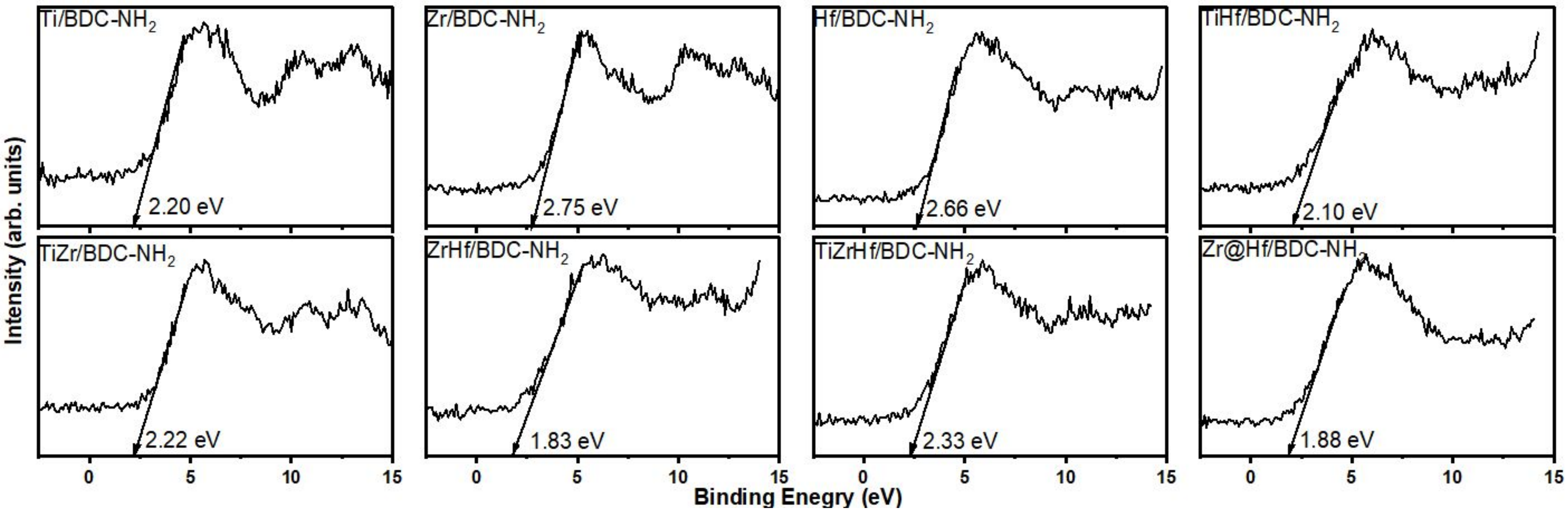

Figure S16. HOCO position calculated from XPS spectra for samples.

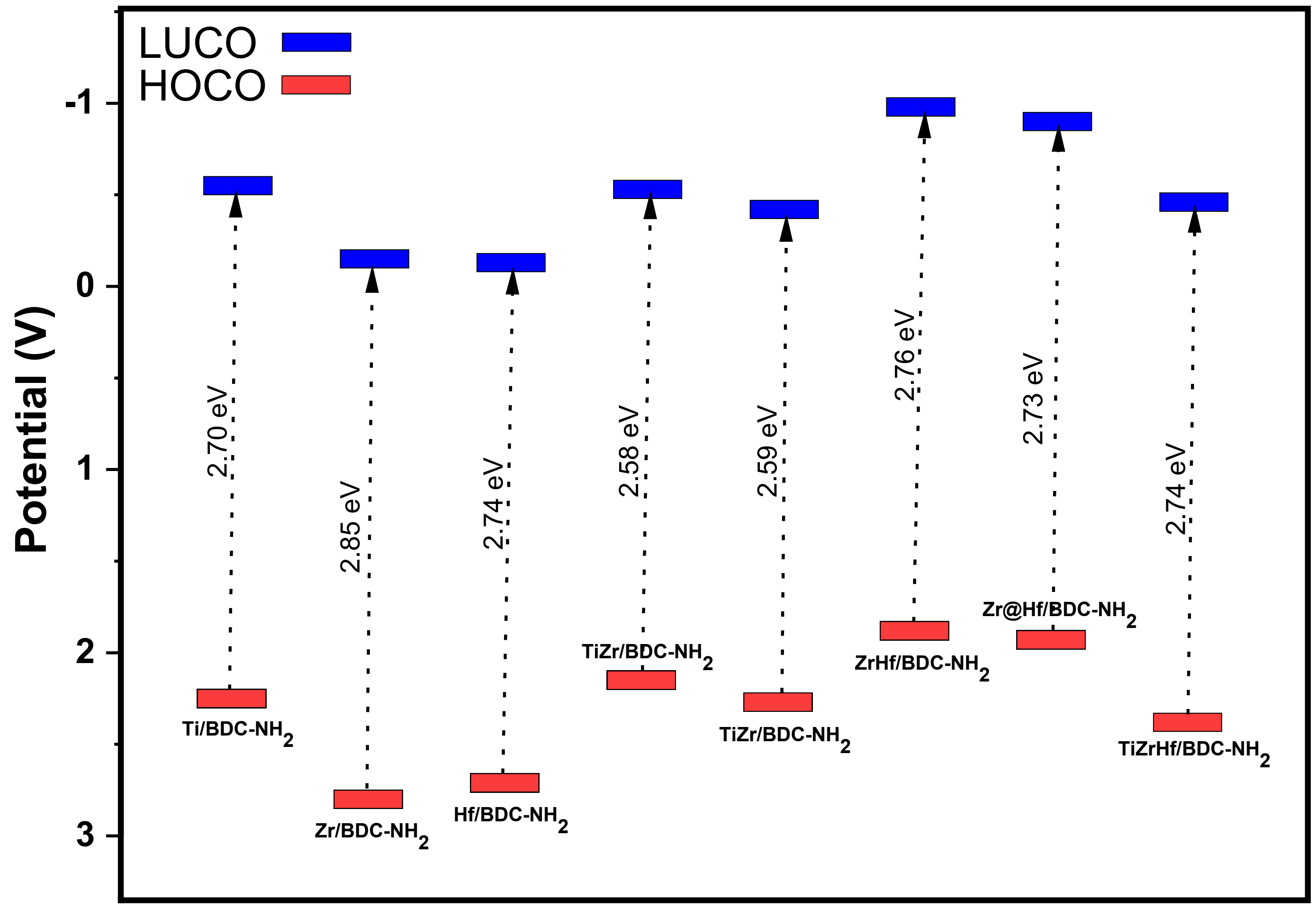

Figure S17. Determined HOCO and LUCO position and the optical gap for the materials obtained (HOCO position determined based on XPS measurements and optical gap based on Tauc plot).

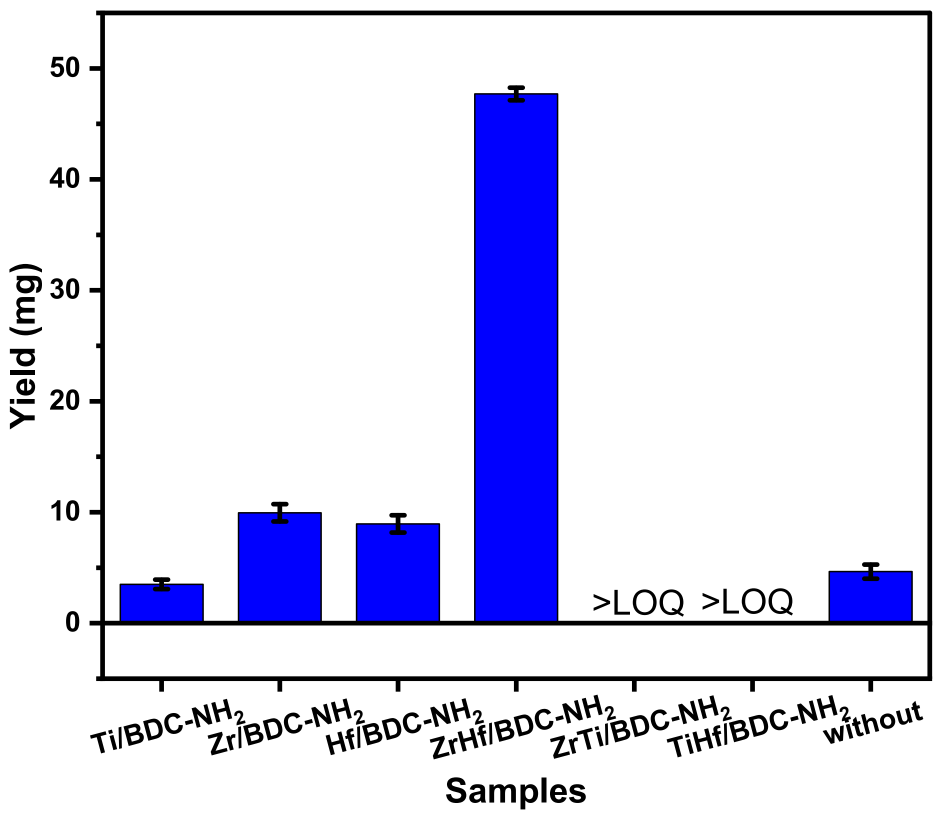

Figure S18. Efficiency of MMA photopolymerization under visible light λ>420 nm with M/BDC-NH_2 samples (12 mg) for 4 h, where M = Ti, Zr, Hf or ZrHf, ZrTi, TiHf.

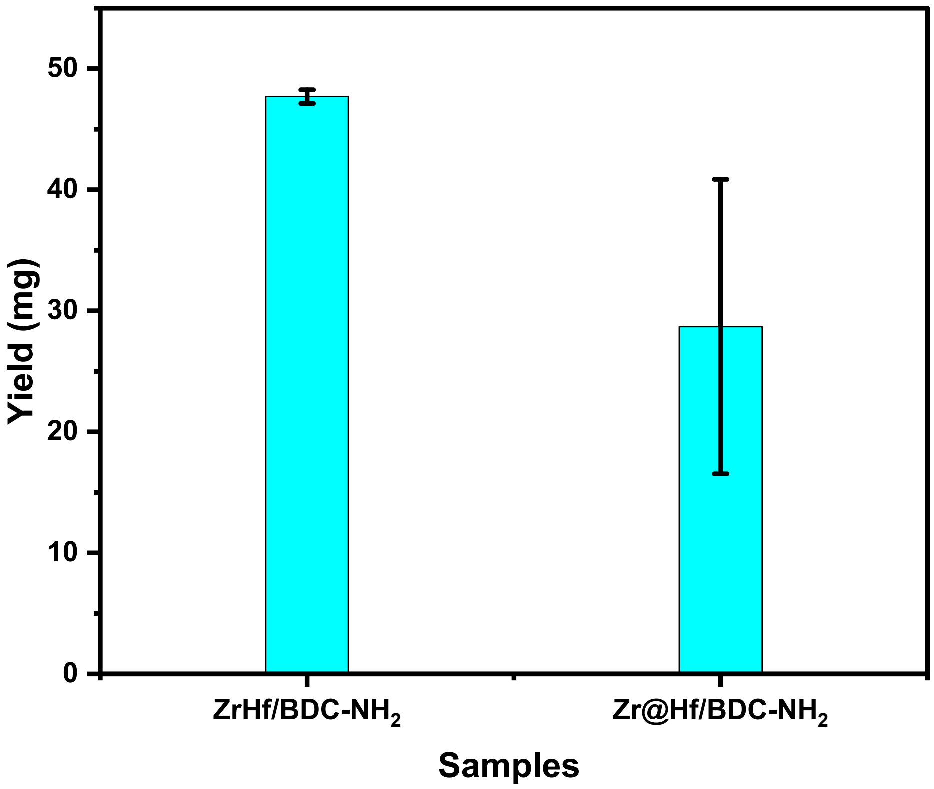

Figure S19. Efficiency of MMA photopolymerization under visible light λ>420 nm with ZrHf/BDC-NH_2 or Zr@Hf/BDC-NH_2 (12 mg) for 4 h.

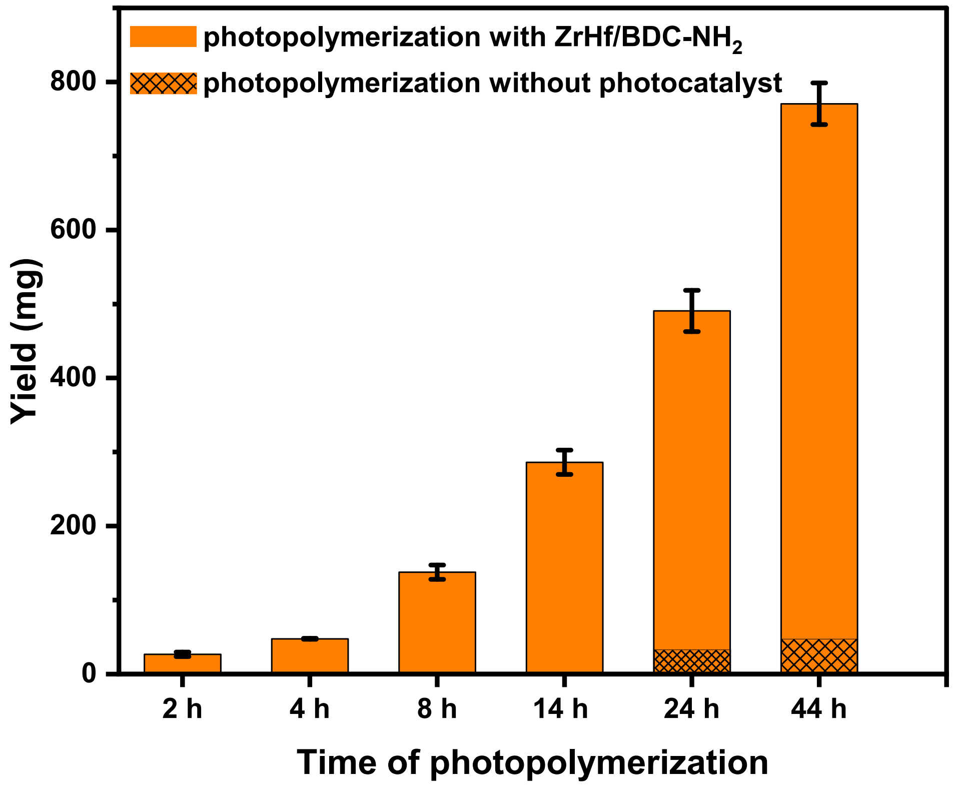

Figure S20. Efficiency of MMA photopolymerization under visible light λ>420 nm with ZrHf/BDC-NH_2 (12 mg) for other time.

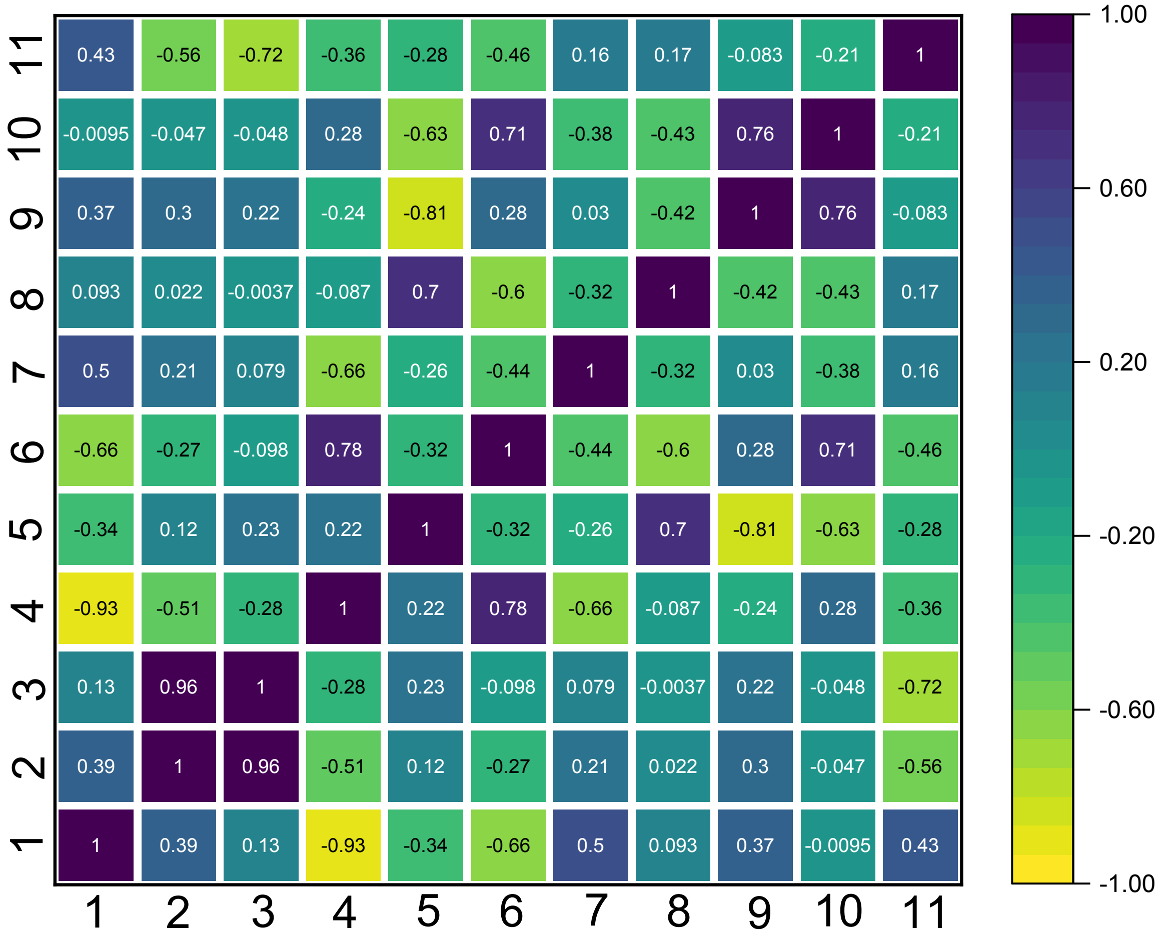

Figure 21. Correlation matrix (Preson method), where (1) – optical gap, (2) HOCO, and (3) LUCO position, (4) absorbance at 420 nm, (5) thermal stability at 200˚C, (6) Ti content (%at, according to XPS), (7) Zr content (%at, according to XPS), (8) Hf content (%at, according to XPS), (9) BET surface area; (10) particle size (11) photopolymerization efficiency.

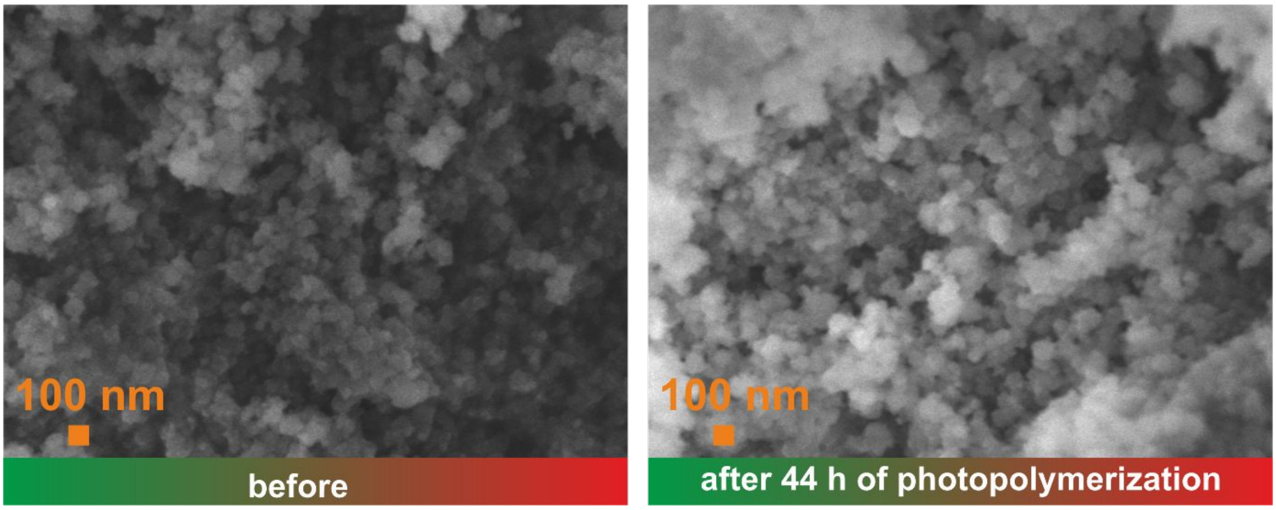

Figure S22. SEM images of ZrHf/BDC-NH_2 before and after 44 h of MMA photopolymerization under visible light (λ>420 nm).

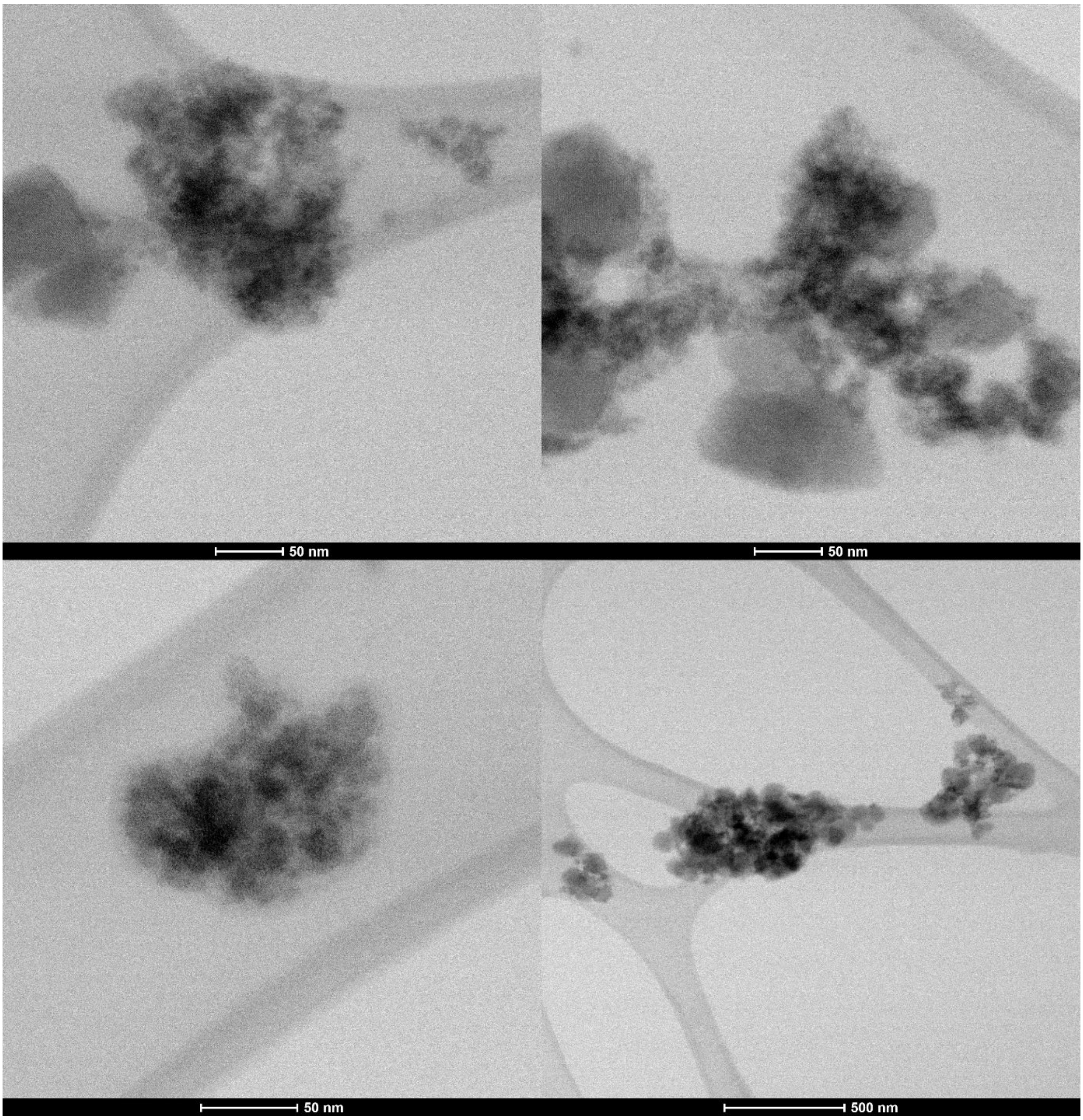

Figure S23. TEM images of ZrHf/BDC-NH_2 after 44 h of MMA photopolymerization under visible light (λ>420 nm).

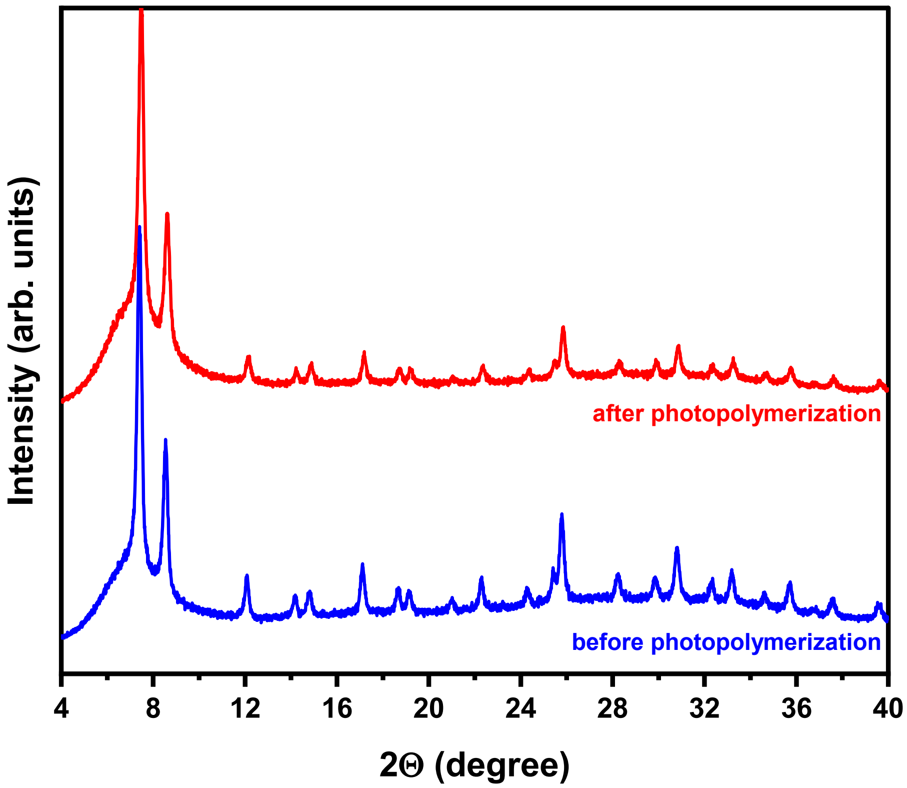

Figure S24. pXRD pattern of ZrHf/BDC-NH_2 after 44 h of MMA photopolymerization under visible light (λ>420 nm).

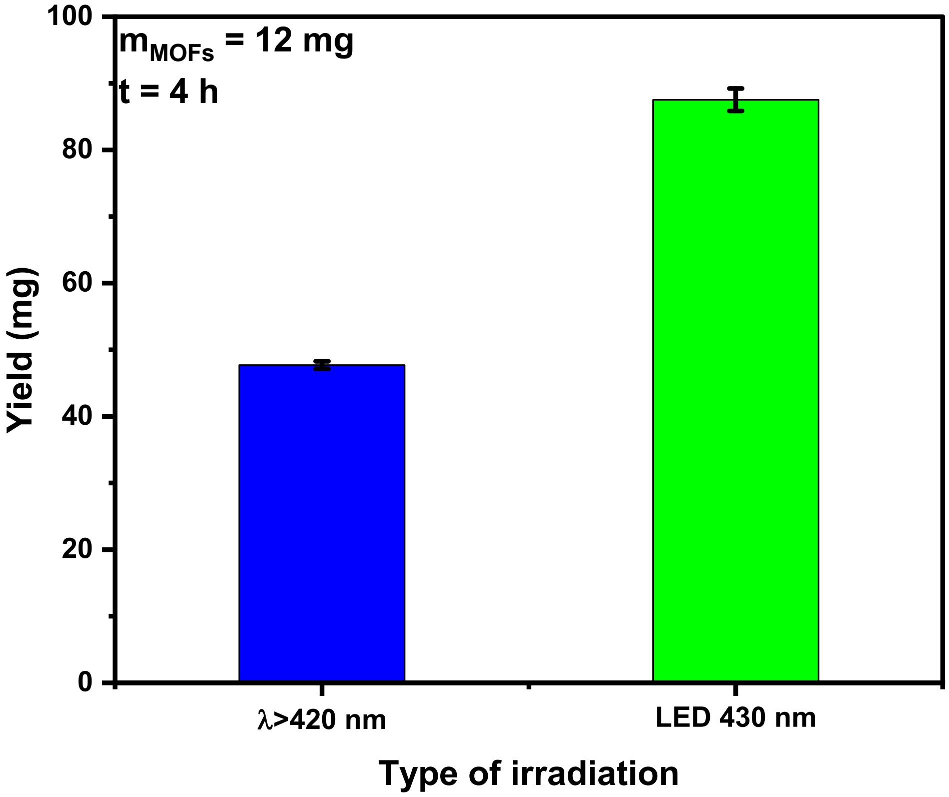

Figure S25. Efficiency of MMA photopolymerization under visible light λ>420 nm or LED 430 nm with ZrHf/BDC-NH_2 (12 mg) for 4h.

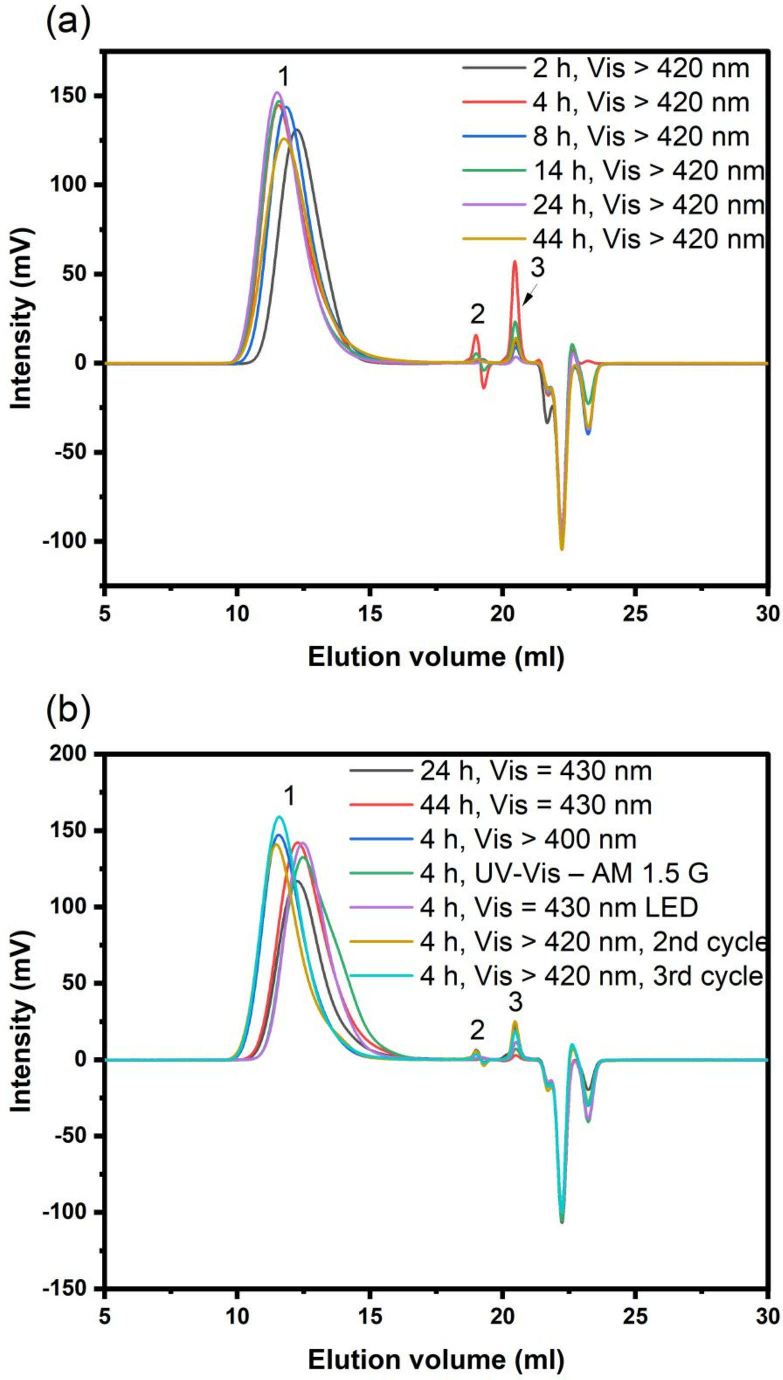

Figure S26. Concentration (DRI) signal comparison of the samples. Peak 1 is high molecular weight polymer. Peak 2 is antioxidant (BHT). Peak 3 is related to the solvent and/or low molecular weight components of the sample formulation. (a) Comparison of DRI chromatograms for samples irradiated with visible light (Vis > 420 nm) showing the effect of irradiation time for durations ranging from 2 to 44 hours. (b) Comparison of signals for samples subjected to various light sources, wavelength ranges, and consecutive reaction cycles to demonstrate the effect of different irradiation conditions.

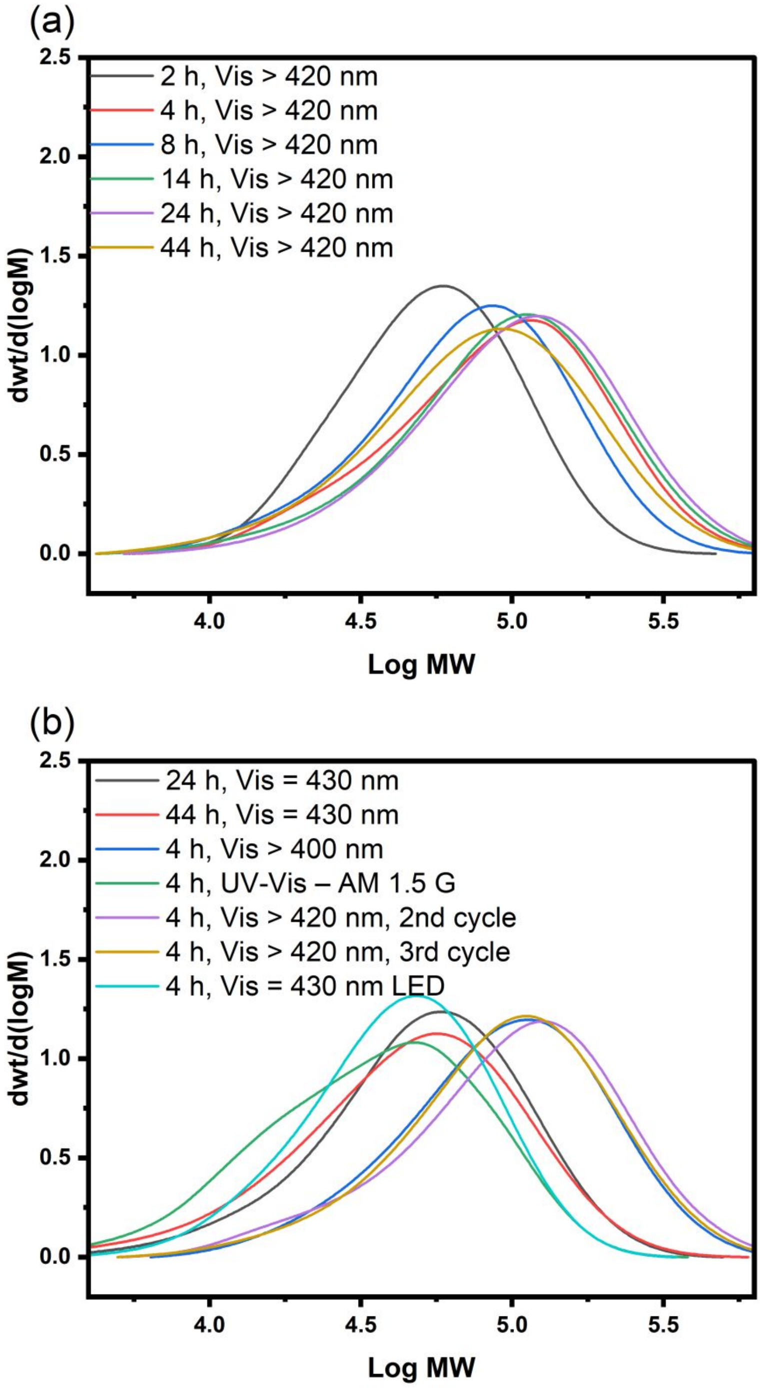

Figure S27. Comparison of differential M_{WD} of the PMMA samples. (a) Comparison of differential molecular weight distribution (MWD) for PMMA samples irradiated at Vis > 420 nm across time intervals of 2, 4, 8, 14, 24, and 44 hours. (b) Differential MWD curves illustrating the impact of specific irradiation parameters, including variation in wavelength, light source, and process stability over the 2nd and 3rd reaction cycles.

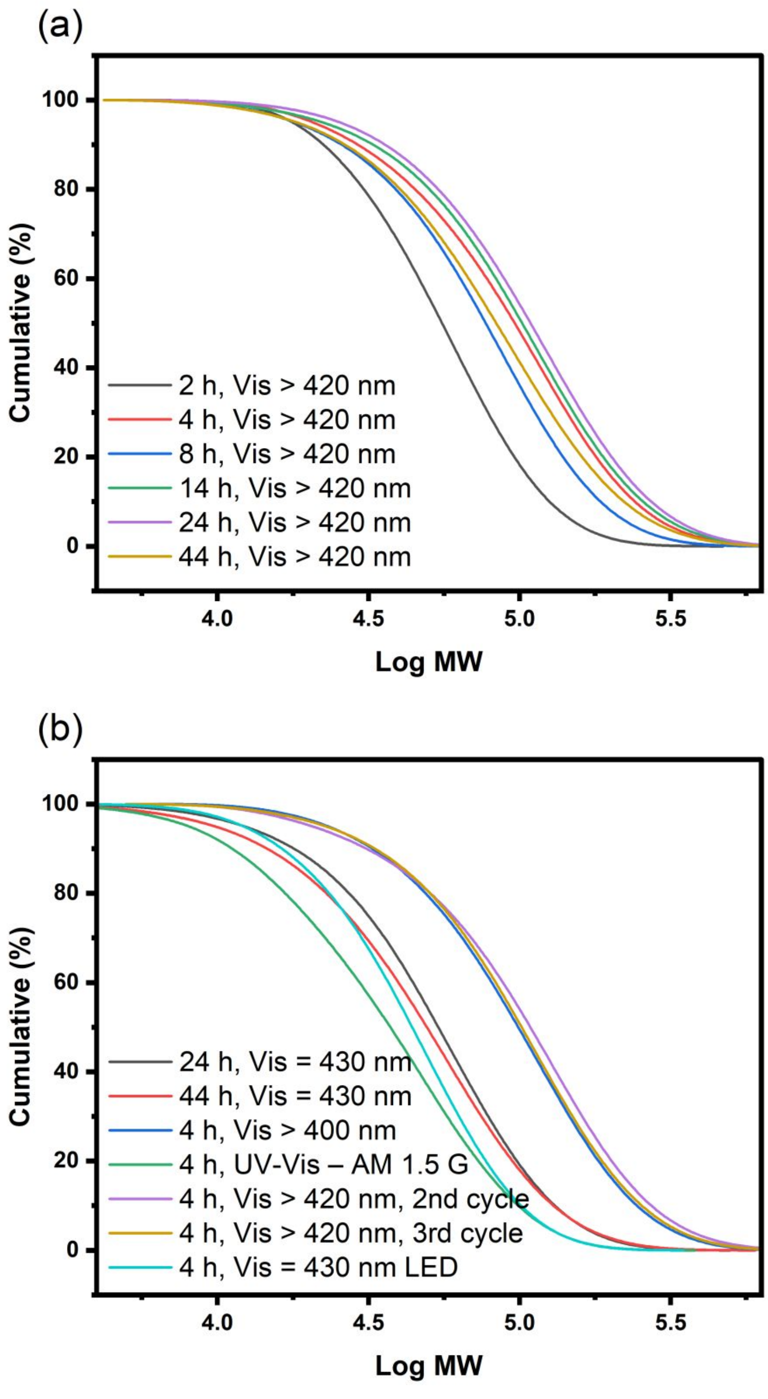

Figure S28. Comparison of cumulative M_{WD} of the PMMA samples. (a) Cumulative molecular weight distribution curves for samples irradiated with visible light as a function of log MW for durations between 2 and 44 hours. (b) Cumulative MWD comparison of PMMA samples prepared under varying experimental conditions, demonstrating the influence of different wavelength filters, light sources, and the consistency of the polymer properties through multiple irradiation cycles.

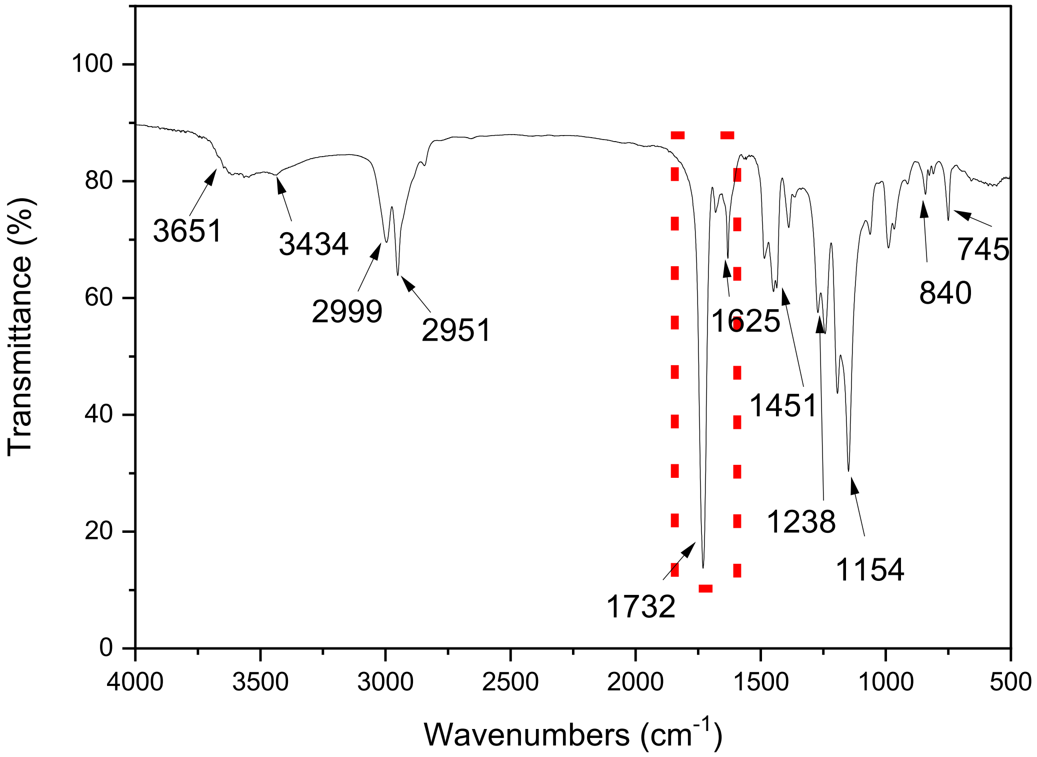

Figure S29. FT-IR spectrum for pMMA obtained using ZrHf/BDC-NH2 and monochromatic irradiation at 430 nm for 24 h.

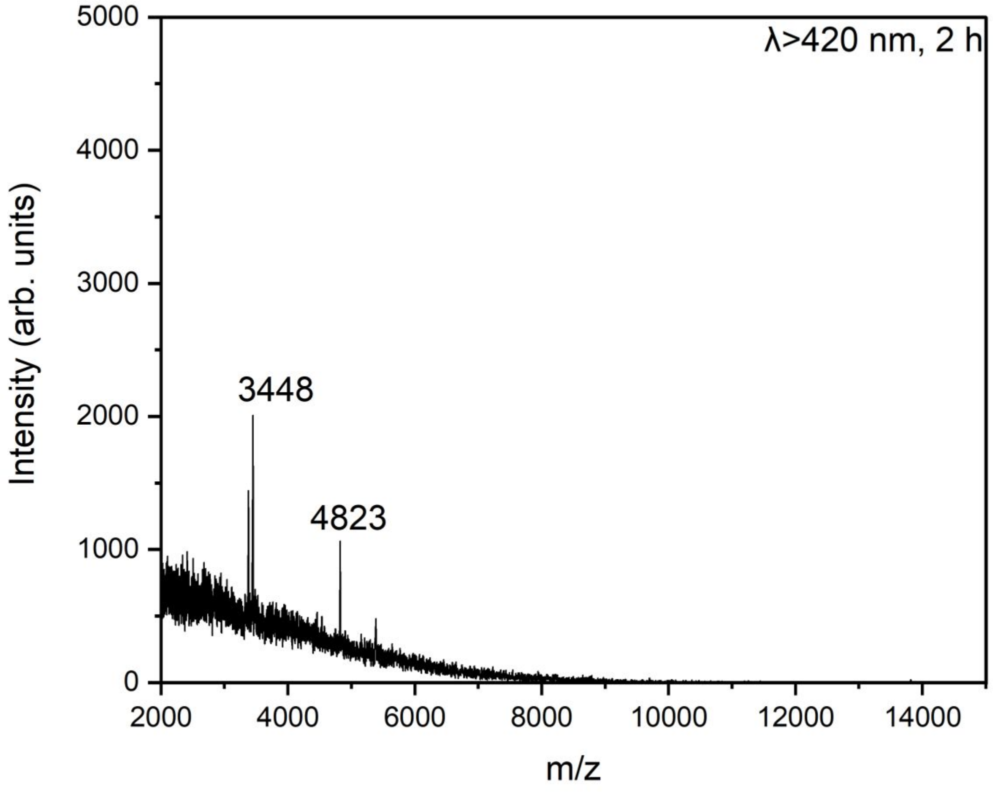

Figure S30. MALDI-TOF-MS of PMMA samples after MMA photopolymerization under visible light λ>420 nm with ZrHf/BDC-NH_2 (12 mg) after 2h.

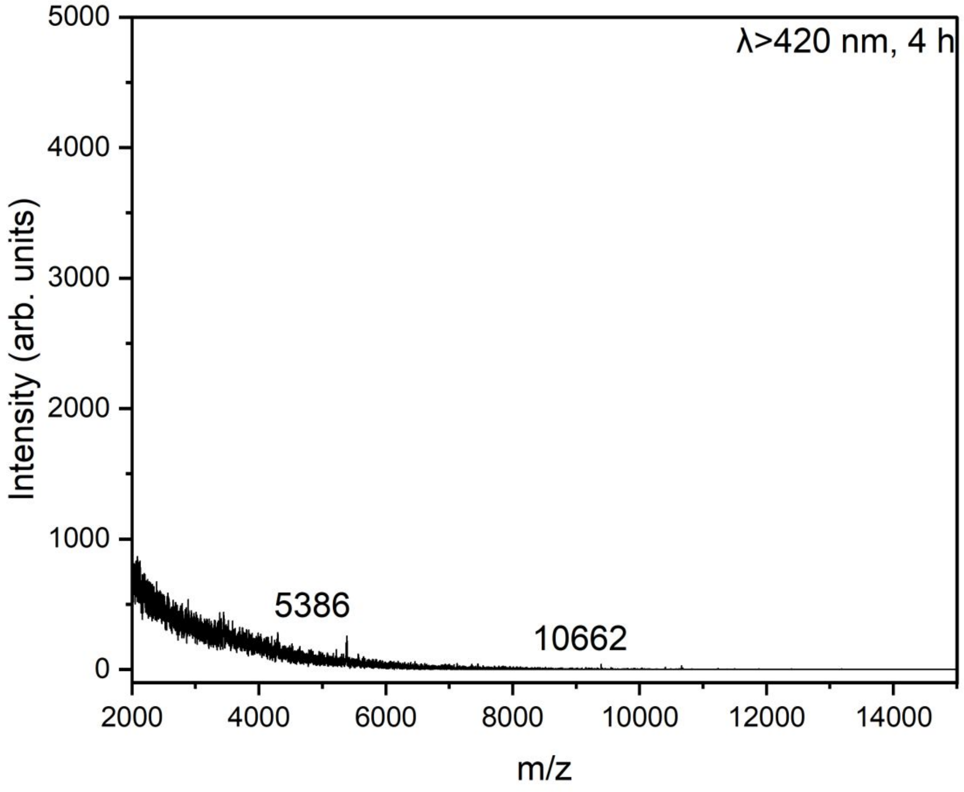

Figure S31. MALDI-TOF-MS of PMMA samples after MMA photopolymerization under visible light λ>420 nm with ZrHf/BDC-NH_2 (12 mg) after 4h.

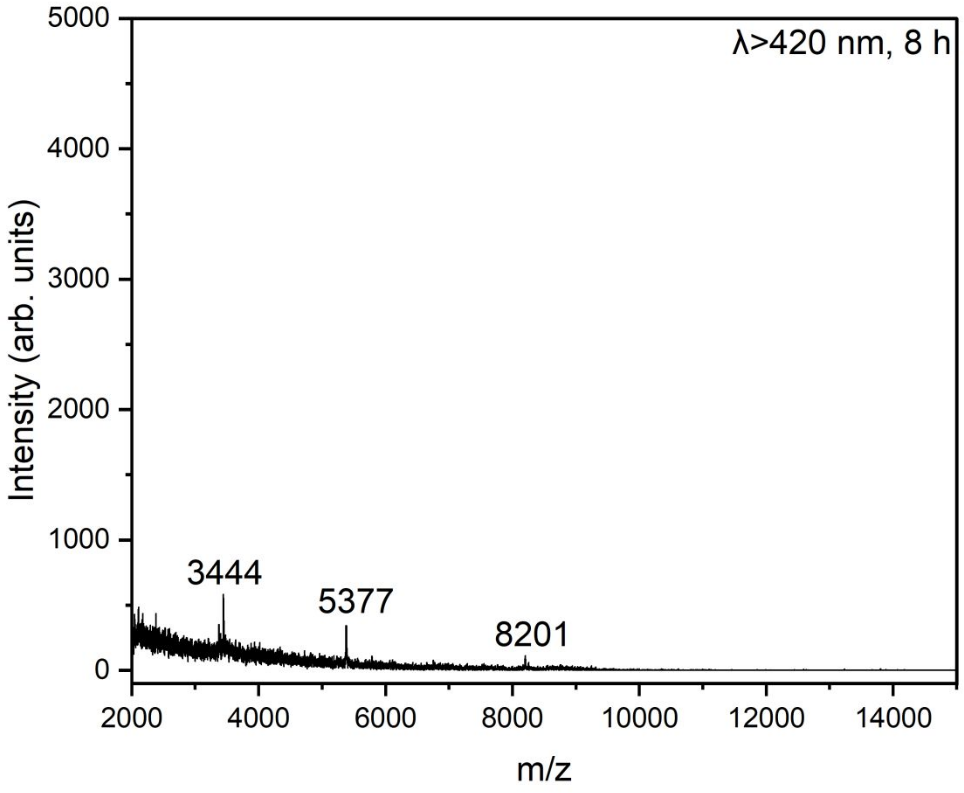

Figure S32. MALDI-TOF-MS of PMMA samples after MMA photopolymerization under visible light λ>420 nm with ZrHf/BDC-NH_2 (12 mg) after 8h.

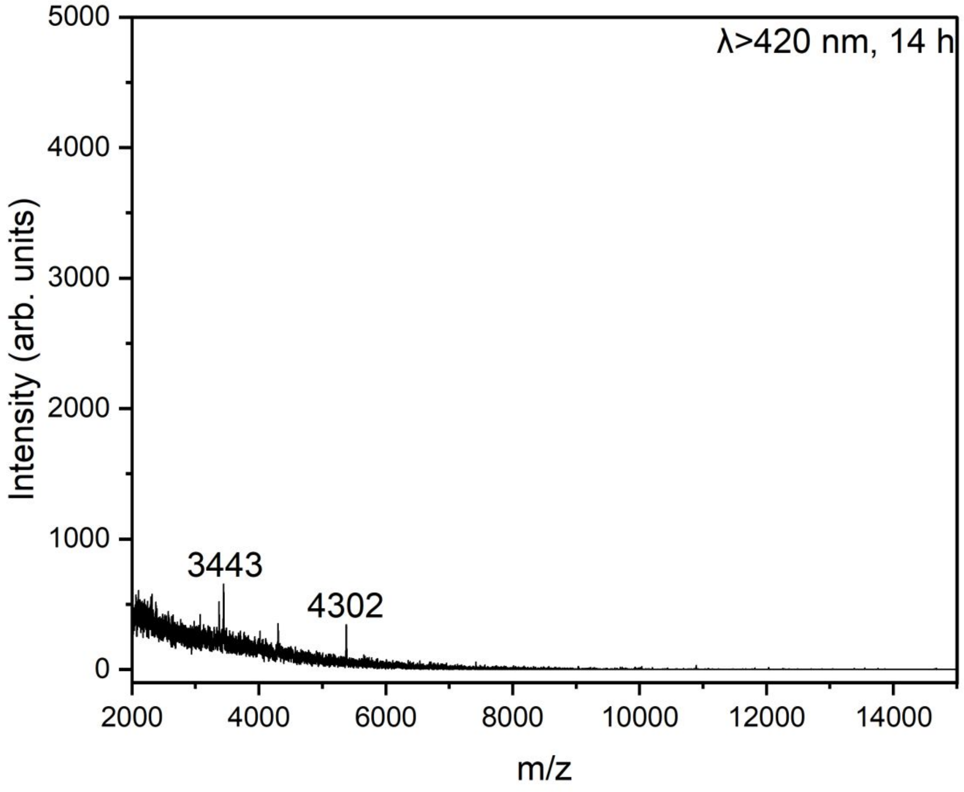

Figure S33. MALDI-TOF-MS of PMMA samples after MMA photopolymerization under visible light λ>420 nm with ZrHf/BDC-NH_2 (12 mg) after 14h.

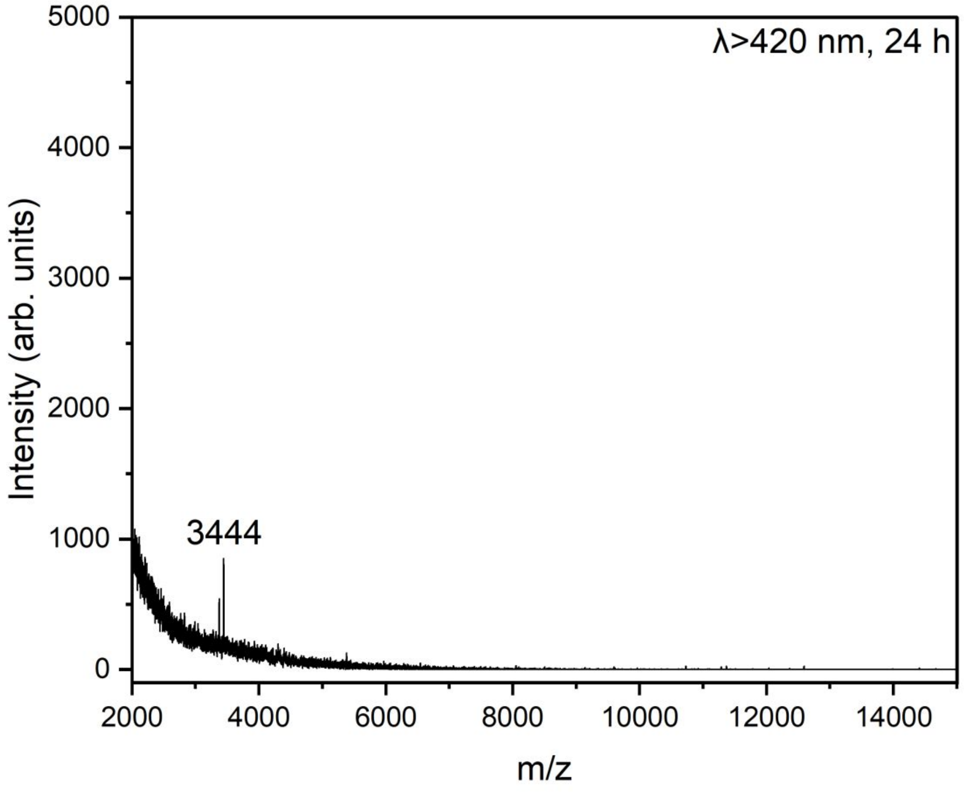

Figure S34. MALDI-TOF-MS of PMMA samples after MMA photopolymerization under visible light λ>420 nm with ZrHf/BDC-NH_2 (12 mg) after 24h.

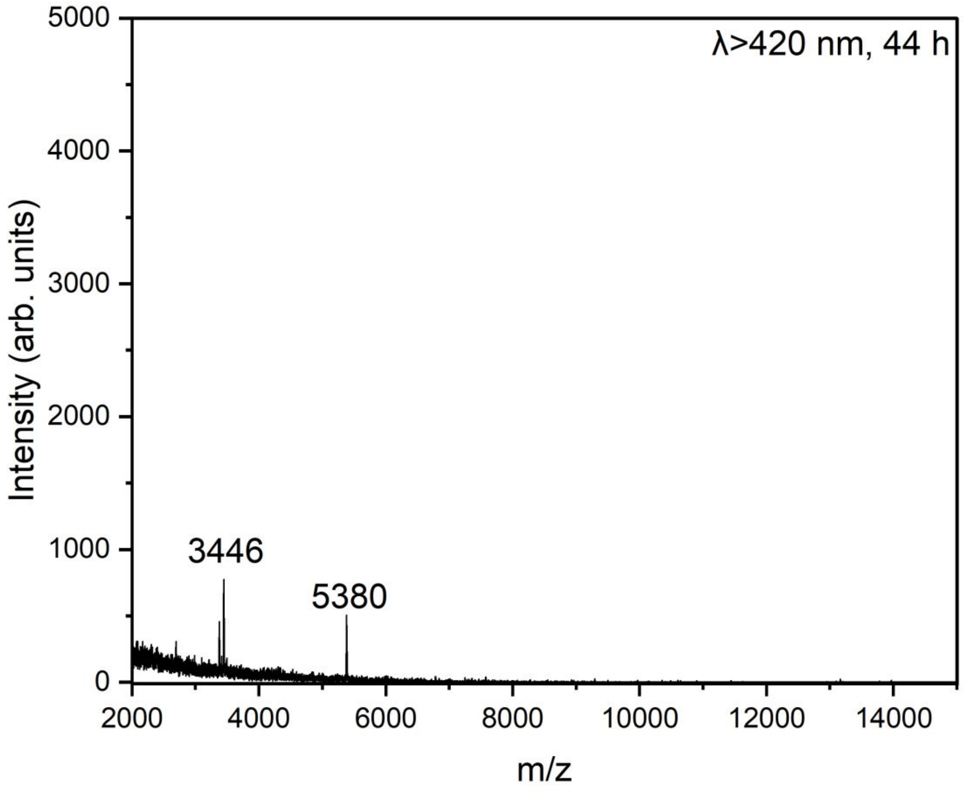

Figure S35. MALDI-TOF-MS of PMMA samples after MMA photopolymerization under visible light λ>420 nm with ZrHf/BDC-NH_2 (12 mg) after 44h.

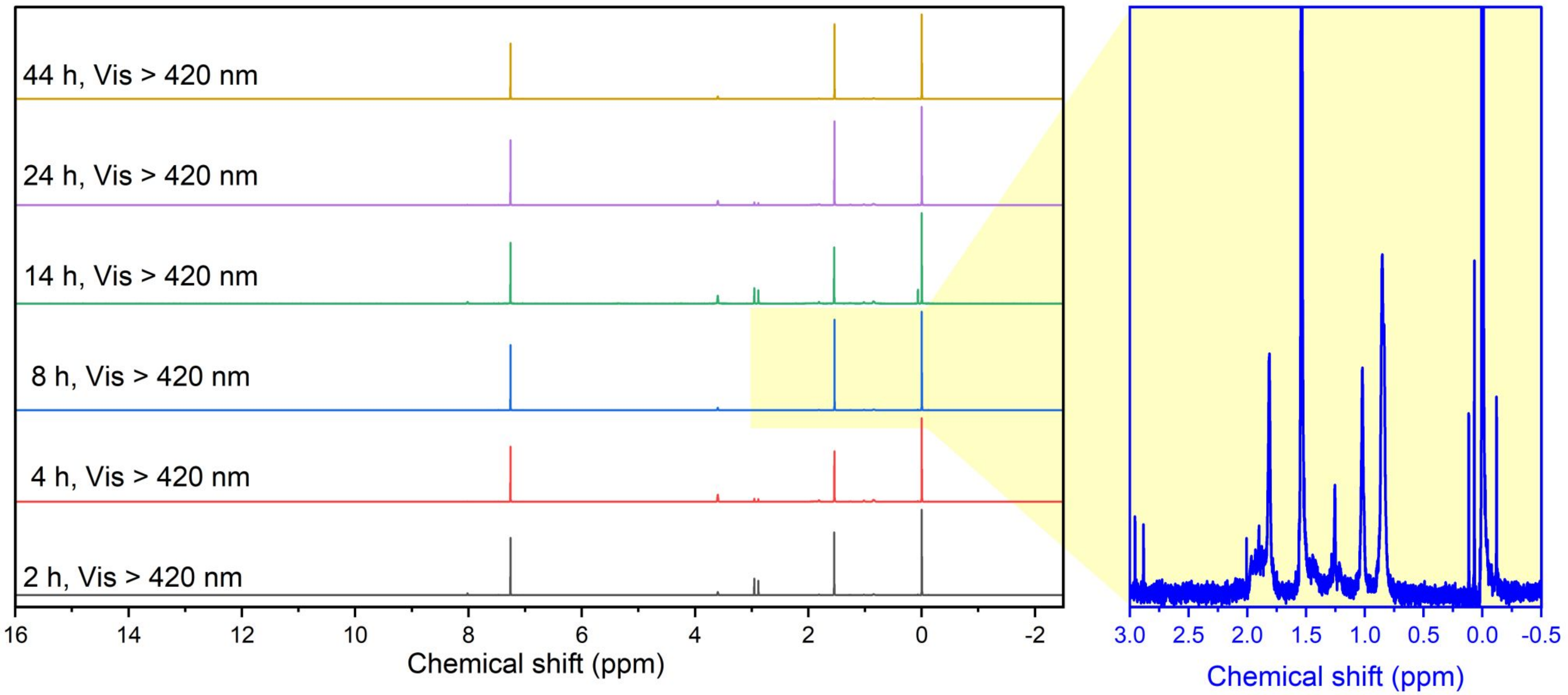

Figure S36. ^{1}H NMR analysis of PMMA samples after MMA photopolymerization under visible light λ>420 nm with ZrHf/BDC-NH_2 (12 mg) for other time.

^{1}H NMR (500 MHz, $CDCl_3$), δ=~ 3.60 (s, ~3H, –OCH_3), ~1.81 (br m, ~2H, CH/CH_2 in main chain), ~1.02–0.85 (m, ~3H, CH_3), ~2.96–2.88 (m, ends of EBPA, CH_2), ~7.26 (s, solvent), 0.00 (s, TMS). ^{13}C NMR (125 MHz, $CDCl_3$, MMA_LED), δ = ~180.07, ~178.27, ~177.15, ~58.74, ~52.42, ~51.82, ~47.24, ~44.39 ppm.

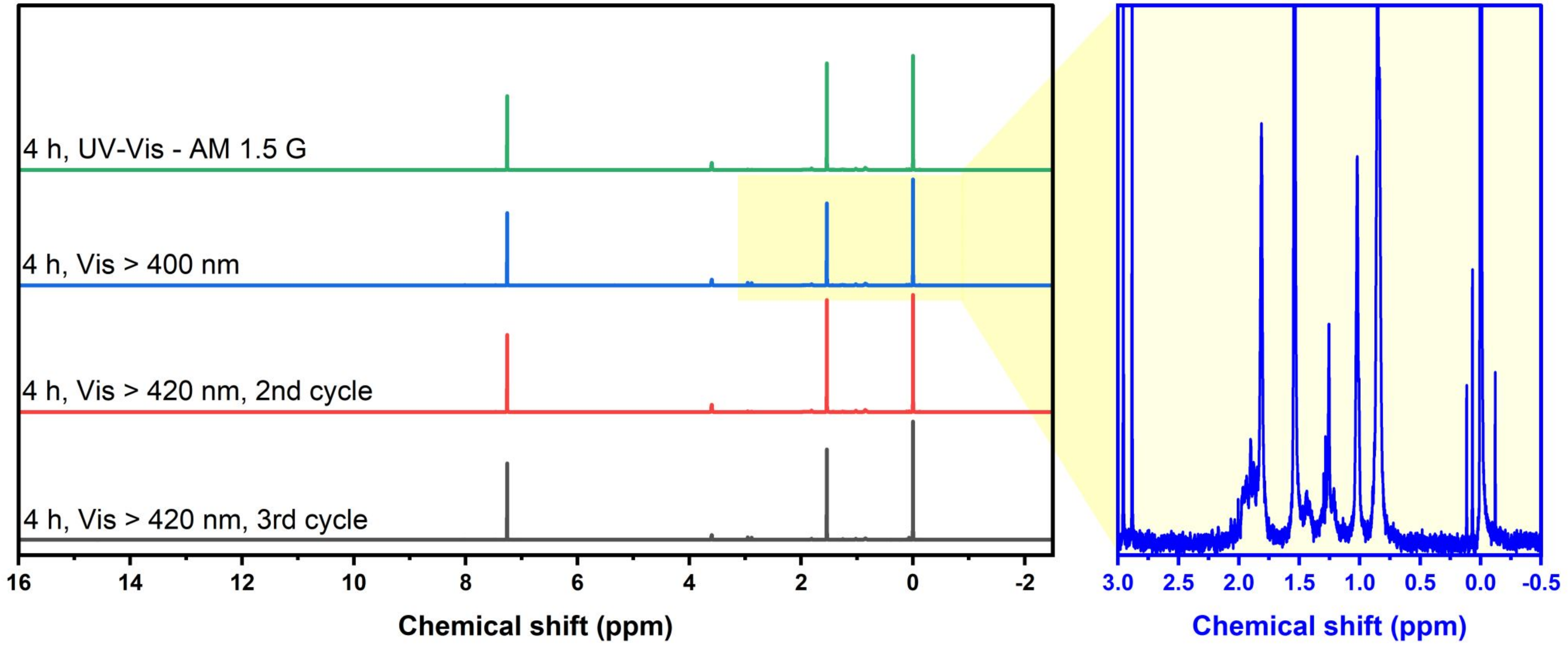

Figure S37. ^{1}H NMR analysis of PMMA samples after MMA photopolymerization under Vis > 420 nm (2nd and 3rd cycles), Vis > 400 nm, and UV-Vis – AM 1.5 G with ZrHf/BDC-NH_2 (12 mg) for 4 h.

^{1}H NMR (500 MHz, $CDCl_3$), δ=~ 3.60 (s, ~3H, –OCH_3), ~1.81 (br m, ~2H, CH/CH_2 in main chain), ~1.02–0.85 (m, ~3H, CH_3), ~2.96–2.88 (m, ends of EBPA, CH_2), ~7.26 (s, solvent), 0.00 (s, TMS). ^{13}C NMR (125 MHz, $CDCl_3$, MMA_LED), δ = ~180.07, ~178.27, ~177.15, ~58.74, ~52.42, ~51.82, ~47.24, ~44.39 ppm.

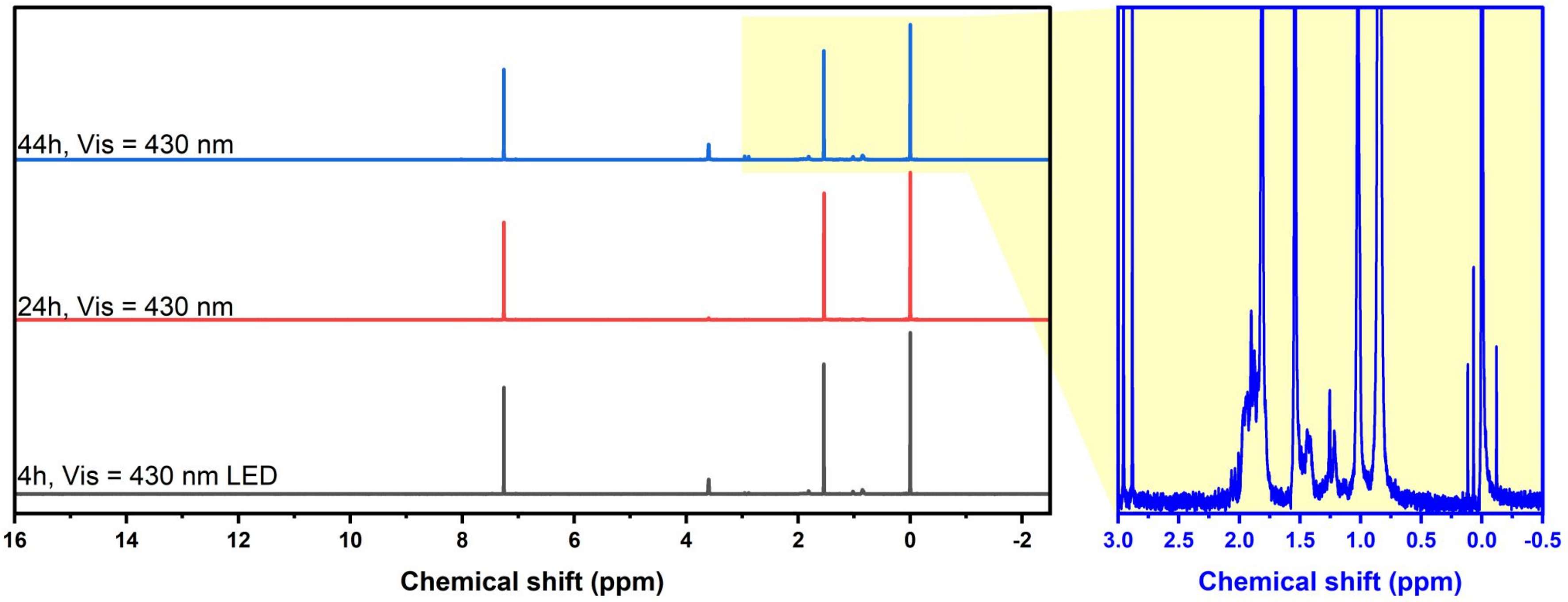

Figure S38. ^{1}H NMR analysis of PMMA samples after MMA photopolymerization under Vis = 430 nm (Xe lamp with monochromatic), and LED with ZrHf/BDC-NH_2 (12 mg) for other time.

^{1}H NMR (500 MHz, $CDCl_3$), δ=~ 3.60 (s, ~3H, –OCH_3), ~1.81 (br m, ~2H, CH/CH_2 in main chain), ~1.02–0.85 (m, ~3H, CH_3), ~2.96–2.88 (m, ends of EBPA, CH_2), ~7.26 (s, solvent), 0.00 (s, TMS). ^{13}C NMR (125 MHz, $CDCl_3$, MMA_LED), δ = ~180.07, ~178.27, ~177.15, ~58.74, ~52.42, ~51.82, ~47.24, ~44.39 ppm.

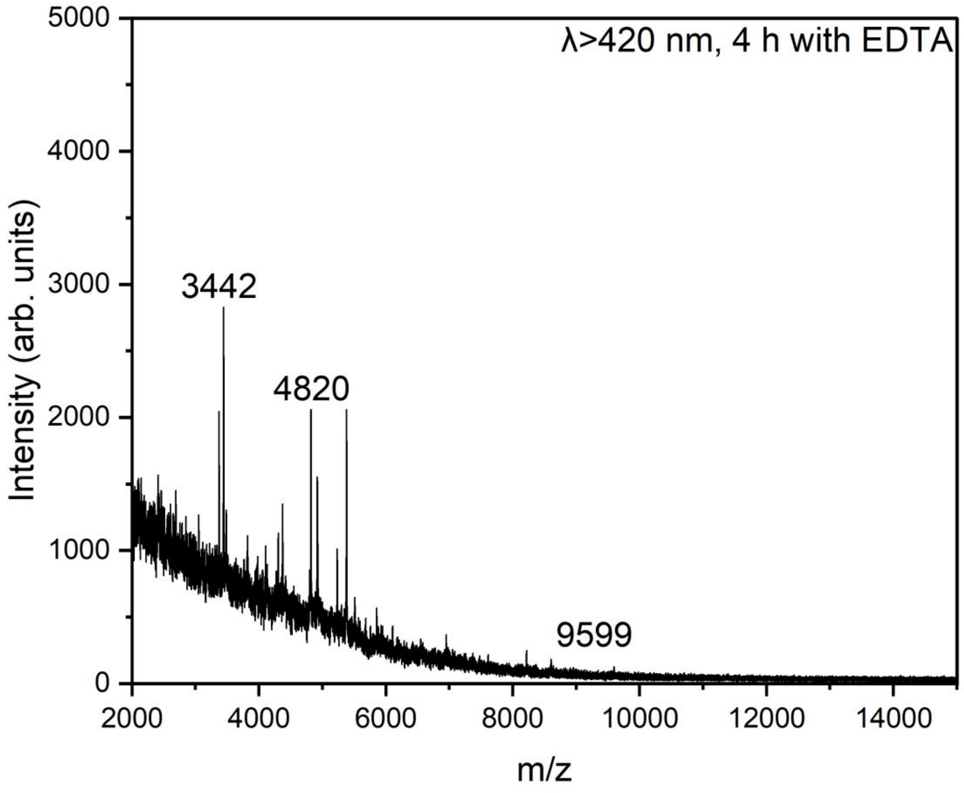

Figure S39. MALDI-TOF-MS of PMMA samples after MMA photopolymerization under visible light λ>420 nm with ZrHf/BDC-NH_2 (12 mg) after 4h with EDTA (5 mM) as scavenger of hole.

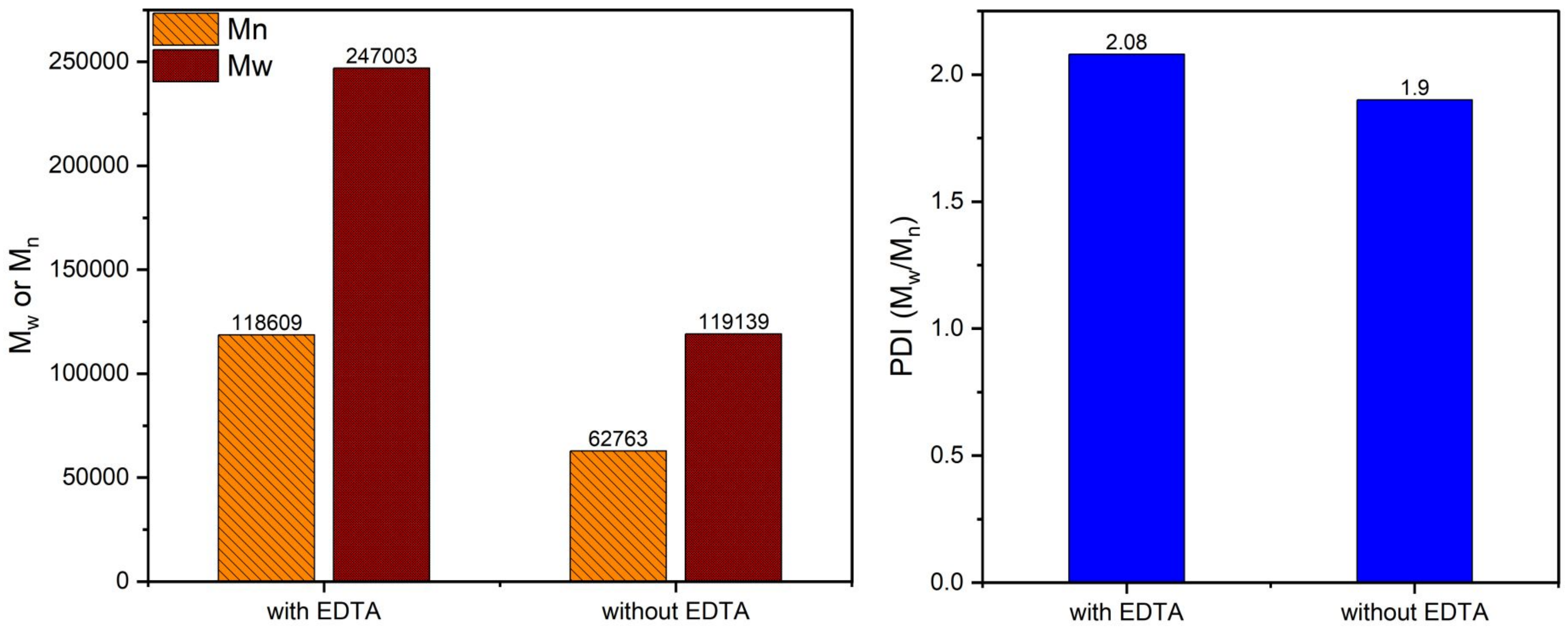

Figure S40. M_w, M_n and PDI index for PMMA samples after MMA photopolymerization under visible light λ>420 nm with ZrHf/BDC-NH_2 (12 mg) after 4h with or without EDTA (5 mM) as scavenger of hole.

Literature:

(1) Nguyen, H. L.; Gándara, F.; Furukawa, H.; Doan, T. L. H.; Cordova, K. E.; Yaghi, O. M. A Titanium-Organic Framework as an Exemplar of Combining the Chemistry of Metal- and Covalent-Organic Frameworks. *J. Am. Chem. Soc.* **2016**, *138* (13), 4330–4333. https://doi.org/10.1021/JACS.6B01233/SUPPL_FILE/JA6B01233_SI_001.PDF.

(2) Hoffman, A. J.; Yee, H.; Mills, G.; Hoffmann, M. R. Photoinitiated Polymerization of Methyl Methacrylate Using Q-Sized ZnO Colloids. *J. Phys. Chem.* **1992**, *96* (13), 5540–5546. https://doi.org/10.1021/J100192A066/ASSET/J100192A066.FP.PNG_V03.

(3) Strandwitz, N. C.; Khan, A.; Boettcher, S. W.; Mikhailovsky, A. A.; Hawker, C. J.; Nguyen, T. Q.; Stucky, G. D. One- and Two-Photon Induced Polymerization of Methylmethacrylate Using Colloidal CdS Semiconductor Quantum Dots. *J. Am. Chem. Soc.* **2008**, *130* (26), 8280–8288. https://doi.org/10.1021/JA711295K/SUPPL_FILE/JA711295K-FILE002.PDF.

(4) Kiskan, B.; Zhang, J.; Wang, X.; Antonietti, M.; Yagci, Y. Mesoporous Graphitic Carbon Nitride as a Heterogeneous Visible Light Photoinitiator for Radical Polymerization. *ACS Macro Lett.* **2012**, *1* (5), 546–549. https://doi.org/10.1021/MZ300116W/SUPPL_FILE/MZ300116W_SI_001.PDF.

(5) Xing, H.; Chen, D.; Li, X.; Liu, Y.; Wang, C.; Su, Z. A Visible-Light Responsive Zirconium Metal–Organic Framework for Living Photopolymerization of Methacrylates. *RSC Adv.* **2016**, *6* (71), 66444–66450. https://doi.org/10.1039/C6RA12134A.

(6) Weng, Z.; Ni, X.; Yang, D.; Wang, J.; Chen, W. Novel Photopolymerizations Initiated by Alkyl Radicals Generated from Photocatalyzed Decarboxylation of Carboxylic Acids over Oxide Semiconductor Nanoparticles: Extended Photo-Kolbe Reactions. *J. Photochem. Photobiol. A Chem.* **2009**, *201* (2–3), 151–156. https://doi.org/10.1016/J.JPHOTOCHEM.2008.10.013.

(7) Momma, K.; Izumi, F. VESTA 3 for Three-Dimensional Visualization of Crystal, Volumetric and Morphology Data. *urn:issn:0021-8898* **2011**, *44* (6), 1272–

1276. https://doi.org/10.1107/S0021889811038970.

(8) *Crystallography Open Database*. https://www.crystallography.net/ (accessed 2025-03-16).
